# New insights into mesoderm and endoderm development, and the nature of the onychophoran blastopore

**DOI:** 10.1186/s12983-024-00521-7

**Published:** 2024-01-25

**Authors:** Ralf Janssen, Graham E. Budd

**Affiliations:** Department of Earth Sciences, Palaeobiology, Uppsala University, Villavägen 16, 75236 Uppsala, Sweden

**Keywords:** Onychophora, Archenteron, Blastopore, T-box transcription factor, Mox, Twist, mef2, Blimp

## Abstract

**Background:**

Early during onychophoran development and prior to the formation of the germ band, a posterior tissue thickening forms the posterior pit. Anterior to this thickening forms a groove, the embryonic slit, that marks the anterior–posterior orientation of the developing embryo. This slit is by some authors considered the blastopore, and thus the origin of the endoderm, while others argue that the posterior pit represents the blastopore. This controversy is of evolutionary significance because if the slit represents the blastopore, then this would support the amphistomy hypothesis that suggests that a slit-like blastopore in the bilaterian ancestor evolved into protostomy and deuterostomy.

**Results:**

In this paper, we summarize our current knowledge about endoderm and mesoderm development in onychophorans and provide additional data on early endoderm- and mesoderm-determining marker genes such as *Blimp*, *Mox*, and the T-box genes.

**Conclusion:**

We come to the conclusion that the endoderm of onychophorans forms prior to the development of the embryonic slit, and thus that the slit is not the primary origin of the endoderm. It is thus unlikely that the embryonic slit represents the blastopore. We suggest instead that the posterior pit indeed represents the lips of the blastopore, and that the embryonic slit (and surrounding tissue) represents a morphologically superficial archenteron-like structure. We conclude further that both endoderm and mesoderm development are under control of conserved gene regulatory networks, and that many of the features found in arthropods including the model *Drosophila melanogaster* are likely derived.

**Supplementary Information:**

The online version contains supplementary material available at 10.1186/s12983-024-00521-7.

## Background

Onychophorans (velvet worms) represent a group of ecdysozoan animals that are closely related with arthropods, although it is still not fully resolved whether they represent the sister group of arthropods or the sister of arthropods + tardigrades (water bears) (recently (reviewed in Wu et al. [1]). Onychophorans, like all bilaterian animals possess three germ layers, the outer ectoderm and the two inner layers, the endoderm and the mesoderm. The outer ectoderm develops early during development from the blastula, but the inner endo- and mesoderm form during gastrulation. Gastrulation is a process by which cells become internalized to form *inter alia* a gastric cavity that the animal uses for food digestion, and thus gastrulation is primarily the origin of the endoderm that significantly contributes to the through-gut. However, in many groups of bilaterian animals, formation of endoderm and mesoderm goes hand in hand [2]. The mode of gastrulation can vary between *invagination* and *epiboly* (the ingrowing of part of the blastula cell sheet that directly leads to the formation of an outer (ectodermal) and inner (endodermal or endodermal/mesodermal) cell sheet), *delamination* (directional cell division of the blastula epithelium), and *immigration* (immigration of cells from one pole (*polar immigration*) or the complete surface of the blastula epithelium) (reviewed in Budd and Jensen [2–4]). The place of organized cell immigration (as seen in *invagination*, *epiboly* and *polar immigration*) is called the blastopore, a structure that is thus crucial for the formation of mesoderm and endoderm.

The origin of the onychophoran mesoderm is relatively well known [5, 6]. In bilaterian animals, mesoderm either forms by schizocoely or enterocoely. Enterocoely describes the direct formation (budding) of mesodermal pouches from the endodermal epithelium, the archenteron. Schizocoely describes the formation of units of mesenchymal tissue between the endoderm and the ectoderm that then each form an internal cavity, the coelom. In a subsequent step these coelomic pouches transform into an epithelial sheet. During schizocoely, typically the somites form in pairs from the posterior of the developing animal, and indeed mesoderm clearly develops by schizocoely in onychophorans: pairs of somites form on either side of the posterior pole of the embryo, the segment addition zone, that then move towards anterior underneath a layer of ectodermal cells (reviewed in Mayer et al. [6]). Together, ectoderm and somites form the early germ band that is initially split by the embryonic slit and surrounding ventral “extra-embryonic tissue” (reviewed in Treffkorn et al. [7]).

The origin of the onychophoran endoderm, however, is less well understood. Early during development, an embryonic slit develops in many groups of onychophorans that closes medially later during development [5, 6, 8–10]. The remaining anterior opening of this slit represents the onychophoran mouth and the remaining posterior opening of the slit represents the anus (thus, this structure is often referred to as the “mouth-anus furrow”). Previous studies investigating morphological and genetic aspects of endoderm development revealed that definite endodermal cells are located predominantly around the embryonic slit but not the posteriorly adjacent posterior pit (the blastopore sensu Manton) (e.g. [5, 8, 10–14]. Expression of endodermal marker genes, however, suggests that definite endodermal cells are already present anterior to the posterior pit and prior to the formation of the embryonic slit [14]. Therefore, it has been suggested that both mesoderm and endoderm derive from the posterior pit and that the endoderm moves into the position around the slit, but does not originate from there [5, 14].

The origin of the endoderm is important because it has a bearing on the whereabouts of the onychophoran blastopore that is generally considered the origin of endoderm development (reviewed in Technau and Scholz [2, 4]). While some authors claim that the slit (or the slit plus the “posterior pit”) represents the blastopore [8, 11, 15, 16], others dispute this idea and instead suggest that only the posterior pit represents the blastopore [5, 10, 17, 18]. If the former is correct, the onychophoran mode of developmental would represent an example of an embryo that gastrulates via amphistomy (the coordinated formation of the mouth and the anus from a slit-like blastopore), and thus would support the amphistomy hypothesis (reviewed in Nielsen et al. [19]).

In this paper, we summarize our current knowledge about mesodermal and endodermal marker gene expression in the onychophoran *Euperipatoides kanangrensis* and provide additional gene expression data on potential endomesodermal, mesodermal and endodermal marker genes. We focus our paper on the T-box family of transcription factors that play important and conserved functions in both determination and separation of endodermal tissue from mesodermal tissue, and the differentiation of mesodermal tissues (reviewed in Showell et al. [20–22]). We identified three previously uninvestigated onychophoran T-box genes that all are expressed in patterns that suggest a conserved function in mesoderm and endoderm development. Beyond that, we also investigated the embryonic expression pattern of the conserved bilaterian mesoderm- and endoderm-marker genes *myocyte enhancer factor-2* (*mef2*), *Mesoderm/Mesenchyme homeobox gene* (*Mox*), *MyoD*/*nautilus* (*nau*), *SoxF*, and *B lymphocyte-induced maturation protein* (*Blimp*) [23–37]. Our data suggest that the gene regulatory networks that underly mesoderm and endoderm development in bilaterian animals are widely conserved also in onychophorans and that differences seen in arthropods likely represent derived features. With respect to the nature of the onychophoran blastopore, we conclude that the posterior pit indeed represents the blastoporal rim (the blastopore sensu* strictu*) and that the embryonic slit represents an “archenteron-like” structure.

## Methods

### Phylogenetic analysis

**Fig. 1:**
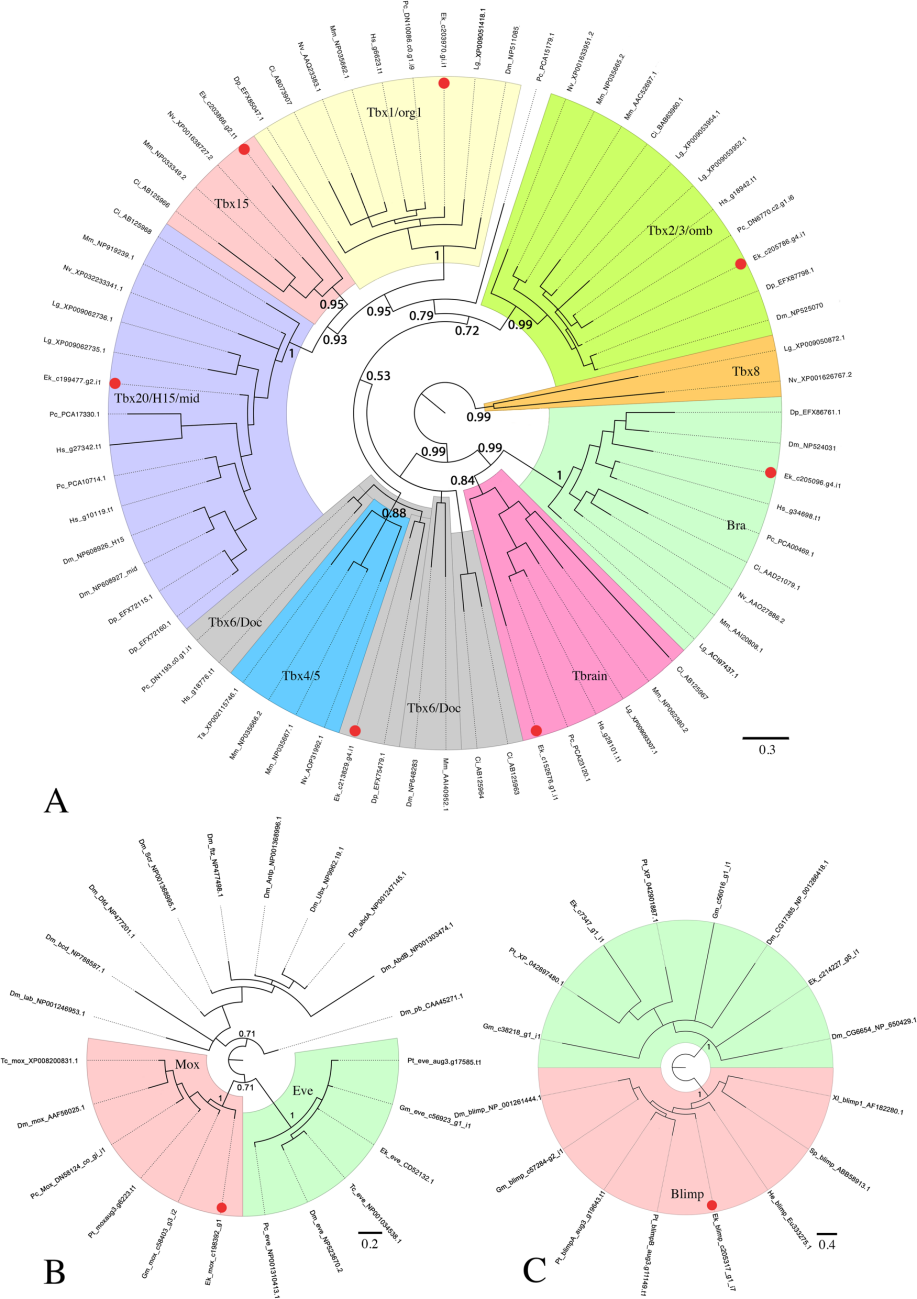
Phylogenetic analyses Phylogenetic trees of T-box genes **A**, Mox genes **B** and Blimp genes **C**. Bayesian analyses using MrBayes [39] applying 0.75 million cycles (T-box Tree), 1 million cycles (Mox tree), and 0.3 million cycles (Blimp tree) for the Metropolis-Coupled Markov Chain Monte Carlo (MCMCMC). The scale bars represent the amino acid substitutions rate per site. Orthologs of Mox proteins are compared with Eve genes and Hox genes **B**. Orthologs of Blimp genes are compared with the most closely related genes of these species, many of them representing previously uncharacterized genes **C**. Support values represent posterior probabilities. Species abbreviations: Ci; *Ciona intestinalis* (Chordata); Dm, *Drosophila melanogaster* (Arthropoda: Insecta); Dp, *Daphnia pulex* (Arthropoda: Branchiopoda); Ek, *Euperipatoides kanangrensis* (Onychophora); Gm, *Glomeris marginata* (Arthropoda: Myriapoda); Hs, *Halicryptus spinulosus* (Priapulida); He, *Hydroides elegans* (Polychaeta); Lg, *Lottia gigantea* (Mollusca); Mm, *Mus musculus* (Vertebrata); Nv, *Nematostella vectensis* (Cnidaria); Pc, *Priapulus caudatus* (Priapulida); *Parasteatoda tepidariorum* (Arthropoda: Chelicerata); Sp, *Strongylocentrotus purpuratus* (Echinodermata); Ta, *Trichoplax adhaerens* (Placozoa); Tc, *Tribolium castaneum* (Arthropoda: Insecta); Xl, *Xenopus laevis* (Vertebrata). Accession numbers are listed in (Additional file 7: Supplementary File 7)

In order to detect gene orthologs in the embryonic transcriptome of the onychophoran *Euperipatoides*, we performed reciprocal BLAST searches (tBLASTn) using protein sequences of known orthologs from the vinegar fly *Drosophila melanogaster* as queries. For the detection of T-box genes we also used previously identified T-box genes from the same species as queries [10, 38]. Protein sequences of the detected onychophoran T-box genes were aligned with T-box genes from other metazoan species using T-Coffee (default parameters, MacVector version 12.6.0) (Nexus files: Additional files 5 and 6: Supplementary Files 5 and 6, Gene identifiers: Additional file 7: Supplementary File 7). We performed phylogenetic analyses with MrBayes [39] as previously described in [40], applying 0.75 million cycles for the Metropolis-Coupled Markov Chain Monte Carlo (MCMCMC) analysis for the main tree (Fig. 1A) and 0.3 million cycles for the tree presented in the (Additional file 1: Supplementary Fig. 1).

*Mox* possesses a homeodomain that is similar to that of *even-skipped* and Hox genes [41]. Therefore, we performed a similar phylogenetic analysis as described above for T-box genes aligning the complete homeodomains of the published Hox and Even-skipped protein from *Drosophila*, the beetle *Tribolium castaneum* and *Euperipatoides* with the homeodomains of *Tribolium* Mox and *Drosophila* Mox (aka *buttonless* (*btn*) (Chiang et al. [42] and the putative *Euperipatoides* Mox protein (Additional file 8: Supplementary File 8). 1 million cycles for the MCMCMC analysis were applied. Mox proteins of *Drosophila*, *Tribolium* and *Euperipatoides* form a monophyletic group with almost total support that represents the sister-group of Eve proteins of these species (Fig. 1B).

We also performed a phylogenetic analysis for the zinc-finger transcription factor Blimp using confirmed orthologs of *Drosophila* and other species plus the best three hits found in the transcriptomes of *Euperipatoides* [9] and the myriapod *Glomeris marginata* [43], and the genomes of the common house spider *Parasteatoda tepidariorum* [44] and *Drosophila* (Additional file 9: Supplementary File 9). 0.3 million cycles for the MCMCMC analysis were applied for this analysis. The here investigated *Euperipatoides* Blimp sequence forms a monophyletic group with confirmed Blimp proteins from other species (Fig. 1C). *mef2* possesses a unique MADS box and Mef2-domain and is thus unique and unlikely to be mistaken for any other distantly related gene in the onychophoran genome [45, 46]. Likewise, *nautilus* (*nau*) possesses gene-specific conserved domains and thus represents another unique gene in animal genomes [23]. Therefore, we did not perform phylogenetic analyses for these two genes.

### PCR, gene cloning, in-situ hybridization, and nuclear staining

Total RNA from a mixed sample of embryos of different developmental stages (all stages previously defined and described in [9] was extracted using TRIZOL (Invitrogen), and reverse transcribed into cDNA using SuperScript IV RT (Invitrogen). Fragment of the identified onychophoran genes were amplified by means of RT-PCR using two sets of gene-specific primers. A nested PCRs was performed using the initial PCR as template. Primer sequences are provided in (Additional file 7: Supplementary File 7). Gene fragments of *Euperipatoides Tbx15-like*, *mef2, * and *nau* were cloned into the PCRII vector (Invitrogen). Fragments of *Euperipatoides Tbrain-like*, *Tbx1/org1*, *Blimp*, and *Mox* were isolated with backwards primers equipped with a T7-promotor sequence overhang [47]. All gene fragments were sequenced on an ABI3730XL automatic sequencer (Macrogen, Seoul, South Korea). PCR products were purified using a PCR purification kit (QIAGEN), purified PCR products of *Tbrain-like*, *Tbx1/org1*, *Blimp*, and *Mox* were used as templates for subsequent probe synthesis with T7 RNA polymerase (ROCHE). Synthesized probes were purified using the RNeasy Kit (QIAGEN). Isolation of *Euperipatoides Tbx6-like*, *twist* (*twi*) *SoxF* and *H15/Tbx20* have been described previously [14, 38, 48]. Whole mount *in-situ* hybridizations (WISH) were performed as described previously [48]. For all genes, we investigated gene expression in embryos of stage 1–21 (staging system as introduced in Janssen and Budd [9]). All relevant expression patterns are presented in this paper. Nuclei were visualized using SYBR Green (incubation of stained embryos in 1:10, 000 SYBR Green in phosphate buffered saline with 0.1% Tween-20 (PBST-0.1%) for 20–30 min).

### Thin sections

After whole mount *in-situ* hybridization, stained embryos of *Euperipatoides* were stepwise dehydrated in a series of 30% ethanol/PBS-T, 50% ethanol/PBS-T, 75% ethanol/PBS-T, 90% ethanol/PBS-T, and 99.5% ethanol/PBS-T; each step 10–15 min at room temperature (RT). Dehydrated embryos were transferred into Xylene and incubated for 10 min at RT. After this first Xylene-incubation, Xylene was exchanged and the embryos were incubated for another 10 min at RT. Xylene was removed and the embryos were transferred into melted paraffin (60 °C). Embryos were incubated in paraffin over night at 60 °C. Embryos were oriented in melted paraffin in small metal containers and transferred onto a cooling plate for approximately two hours. Embedded samples were then stored overnight (or until sectioning) in a freezer at − 20 °C. The paraffin-embedded stained embryo samples were sectioned using a Leica RM2155 Microtome. Thin sections of 6 µm were produced and transferred with a brush to the surface of a water bath (37 °C) for relaxation. Relaxed thin sections were transferred to frosted microscope glass slides. Sections were dried over night at 37 °C. After drying, the slides were deparaffinized in staining jars twice for 10 min in Xylene, and were subsequently transferred to glass jars with Clear-Rite 3 (Epredia™, 6901). To mount the samples, few drops of VectaMount® Permanent Mounting Medium (Vector Laboratories, H-5000) were applied to the slides before covering them with a coverslip. The slides were left to dry on a fume bench for one to four hours. The edges of the cover slips were sealed and fixed with nail polish.

### Data documentation

Photographing of stained embryos including the detection of the nuclear dye SYBR green and thin-sectioned embryos were performed under a MZ-FLIII Leica dissection microscope equipped with a Leica DC490 digital camera and an external UV-light source. Whenever indicated, linear adjustments were performed on colour, contrast and brightness using the image-processing software Adobe Photoshop 2022. The phylogenetic trees were made using FigTree V1.4.4.

## Results

### Phylogenetic analysis of T-box genes

In previous studies, we published the sequences and embryonic expression patterns of four *Euperipatoides* T-box genes, *optomotor-blind1* (*omb1*) (ID: c213829; HG326421), *optomotor-blind2* (*omb2*) (ID: c205786; HG326422), *Tbx20*/*H15* (ID: c199477; HG326423) [38], and *brachyury* (*bra* (ID: c205096, LN812023 [10].

While orthology of *Tbx20*/*H15*, *bra*, and *omb2* was confirmed, the newly performed phylogenetic analysis revealed that the previously described *omb1* gene does not represent an ortholog of *omb* but likely represents the ortholog of *Tbx6/dorsocross* (*doc*) (Fig. 1A). In this context, it should be stressed that this class of T-box genes usually does not form a well-supported monophyletic group in phylogenetic analyses [49–52]. Likewise, in our analyses, *Tbx6*/*doc* genes, including the putative onychophoran *Tbx6/doc* gene, do not form a monophyletic group (Fig. 1A and Additional file 1: Supplementary Fig. 1) but fall together with *Tbx4/5* orthologs, a group of T-box genes that has been secondarily lost in many animal lineages (reviewed in Sebé-Pedrós and Ruiz-Trillo [53]) (Fig. 1A). Orthology of the onychophoran *Tbx6/doc* gene, however is likely because all other identified T-box genes, including the newly discovered *Tbx1/org1, Tbrain/Eomes, * and *Tbx15*, fall into monophyletic groups, and thus the gene in question likely does not represent an ortholog of any of these classes of T-box genes (Fig. 1A). The previously described *omb2* gene [38] thus represents the only identified *omb* gene in the onychophoran, therefore hereafter simply referred to as *omb* (Fig. 1A).

Our analysis further revealed that the onychophoran possesses at least three more T-box genes that cluster with *Tbx1*/*optomotor-blind-related1* (*org1*) orthologs (ID: c203970), *Tbx15* orthologs (ID: c203866), and *Tbrain/Eomes* orthologs (ID: c152676) from other metazoan species (Fig. 1A). The onychophoran *Tbx1/org1* gene clusters with total support with confirmed orthologs of *Tbx1/org1* genes from other metazoan species (Fig. 1A).

The putative onychophoran *Tbx15* gene clusters with fidelity with other metazoan *Tbx15* genes (Fig. 1A). While the related *Tbx20/H15* and *Tbx1/org1* genes are present in most metazoans, *Tbx15* genes appear to have been lost in many groups of animals (reviewed in Sebé-Pedrós and Ruiz-Trillo [53]). Retention of *Tbx15* has been described for the water flea *Daphnia pulex* and the centipede *Strigamia maritima* [53], their supplementary data). We tried to identify these sequences, but unique sequence identifiers were not supplied in the aforementioned publication, and they are not part of the initial study conducted by the same authors [52], their supplementary data). We were also unable to identify any arthropod *Tbx15* orthologs by our own analyses including the water flea *Daphnia*, the spider *Parasteatoda* and the millipede *Glomeris* (Additional file 1: Supplementary Fig. 1). The discovery of a putative *Tbx15* gene in the onychophoran is thus quite surprising as it appears to be the first report of a retained *Tbx15* ortholog in any ecdysozoan species (note that there is neither a *Tbx15* gene in the nematode worm *Caenorhabditis elegans* [54] nor in the priapulids *Priapulus caudatus* and *Halicryptus spinulosus* that represent a group of basally branching ecdysozoans [55] (Fig. 1A)). This onychophoran T-box gene, however, branches basally in its monophyletic group, and thus its orthology with confirmed *Tbx15* genes from other species cannot be confirmed beyond doubt. It is possible that this gene represents a derived ortholog of *Tbx15*, or a *Tbx15*-like orphan T-box gene.

To our knowledge, there is no identified ortholog of *Tbrain* in any panarthropod species [53]. One of the newly identified onychophoran T-box sequences, however, is most similar to *Tbrain* genes from other metazoan animals, but like for *Tbx15*, the onychophoran sequence is basally branching within this relatively well-supported monophyletic group (Fig. 1A). It is therefore nevertheless possible that the gene represents a derived *Tbrain* ortholog or a *Tbrain*-like orphan rather than a true ortholog of *Tbrain*.

Because the phylogenetic tree of T-box genes is not resolved beyond doubt with respect to the identity of the putative onychophoran orthologs of *Tbx6*, *Tbx15* and *Tbrain*, we cautiously designated these onychophoran genes as *Tbx6-like*, *Tbx15-like*, and *Tbrain-like*. Interestingly, however, the expression patterns of these three onychophoran genes are very much comparable with the expression patterns of confirmed *Tbx6*, *Tbx15*, and *Tbrain* genes in other bilaterian animals, and this indeed may be interpreted as additional support for their orthology with these genes.

### Expression of onychophoran T-box genes

**Fig. 2:**
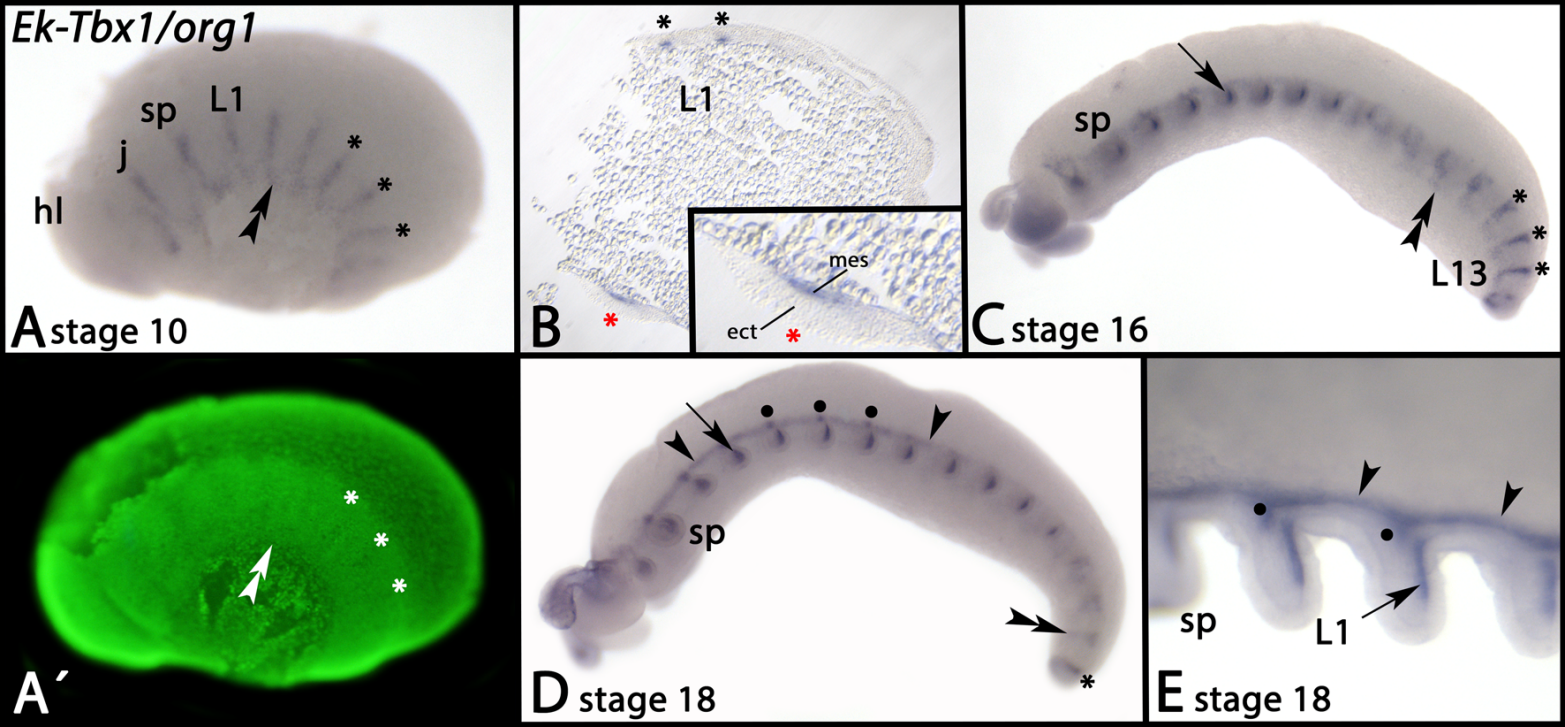
Expression of *Tbx1/org1* In all panels, anterior is to the left. Panels A, C and D represent lateral views, dorsal up. Panel B represents a thin section. Panel E represents a dorsal view. Panel A´ represents a SYBR Green staining of the embryo shown in panel A. The double arrowheads in panels A and C point to ventral expression. The asterisks in panels A-D mark segmental expression in newly formed segments. The red asterisk in B marks the enlarged region shown in the inlay in B. The arrow in panels C and E point to expression in the limbs. Arrowheads in panels D and E point to dorsal expression. Filled circles in panel D and E mark patches of stronger expression dorsal to each appendage. Developmental stages are indicated (staging system after [9], their supplementary data). Abbreviations: ect, ectoderm; hl, head lobe; j, jaw; L, leg; mes, mesoderm; sp, slime papilla

*Tbx1/org1* is first expressed in the form of transverse segmental stripes (Fig. 2A, asterisks) and a transient diffuse pattern in the most ventral region of the developing germ band (Fig. 2A, double-arrowhead). The latter could be associated with the developing ventral nervous system. The former segmental expression is in the mesoderm (Fig. 2B). At later developmental stages, both the segmental mesodermal stripes and the diffuse ventral expression disappear from older (i.e. more anterior) segments (Fig. 2C, D). The segmental transverse stripes of expression either disappear or transform into patches of expression in the developing appendages (Fig. 2C-E). Expression in the appendages is also mesodermal, but restricted to the posterior of the appendages (Fig. 2C-E, arrows). At later developmental stages (and more anterior/older segments), expression also appears dorsal to the appendages (Fig. 2D, E, arrowheads). Within this continuous domain of expression, there is a patch of stronger expression dorsal to each appendage, except for the jaws (Fig. 2D, E, filled circles).

**Fig. 3:**
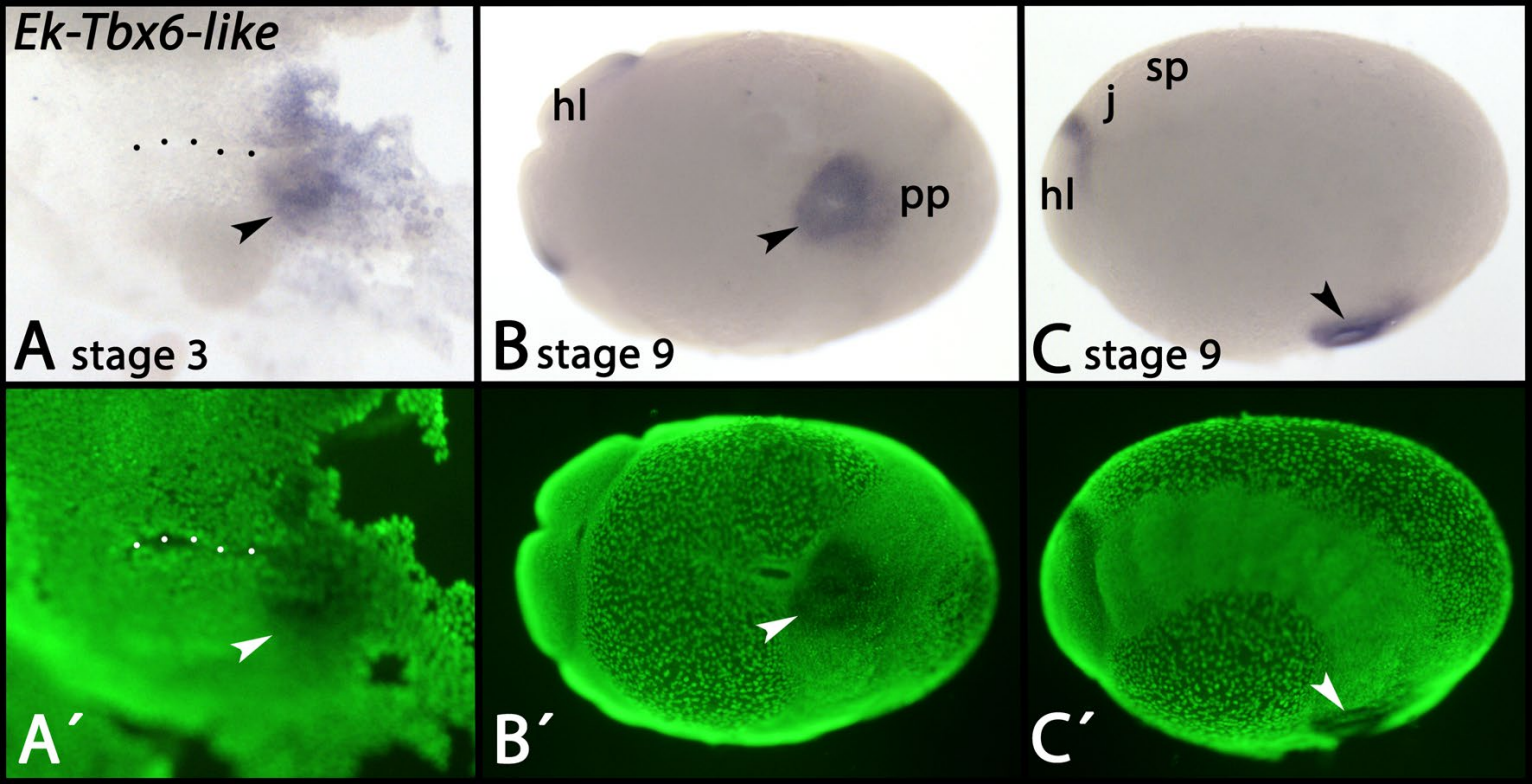
Expression of *Tbx6-like* In all panels, anterior is to the left. Panels A and B represent ventral views. Panel C represents a lateral view, dorsal up. Panels A´-C´ represent SYBR Green staining of the embryos shown in panels A-C. The dotted line in panel A marks the embryonic slit. The arrowheads in panels A-C point to expression in the ectoderm of the posterior pit. Developmental stages are indicated (staging system after [9], their supplementary data). Abbreviations: hl, head lobe; j, jaw; pp, posterior pit; slime papilla

Expression of *Tbx6-like* appears early during germ band formation in the posterior pit region (Fig. 3A–C, arrowheads). Later during development, expression appears dorsally in the head lobes (Fig. 3B, C) and all appendages (Additional file 2: Supplementary Fig. 2A, B, double-arrowheads) (also see [38]. Expression in the posterior pit remains during development (Additional file 2: Supplementary Fig. 2A, arrowhead). At late developmental stages, expression is also in the dorsal extra-embryonic tissue (Additional file 2: Supplementary Fig. 2A, B, asterisks). This aspect of *Tbx6-like* expression is thus possibly conserved between onychophorans and insects in which *Tbx6/doc* genes are expressed in the extra-embryonic membranes (reviewed in Panfilio et al. [56]).

**Fig. 4:**
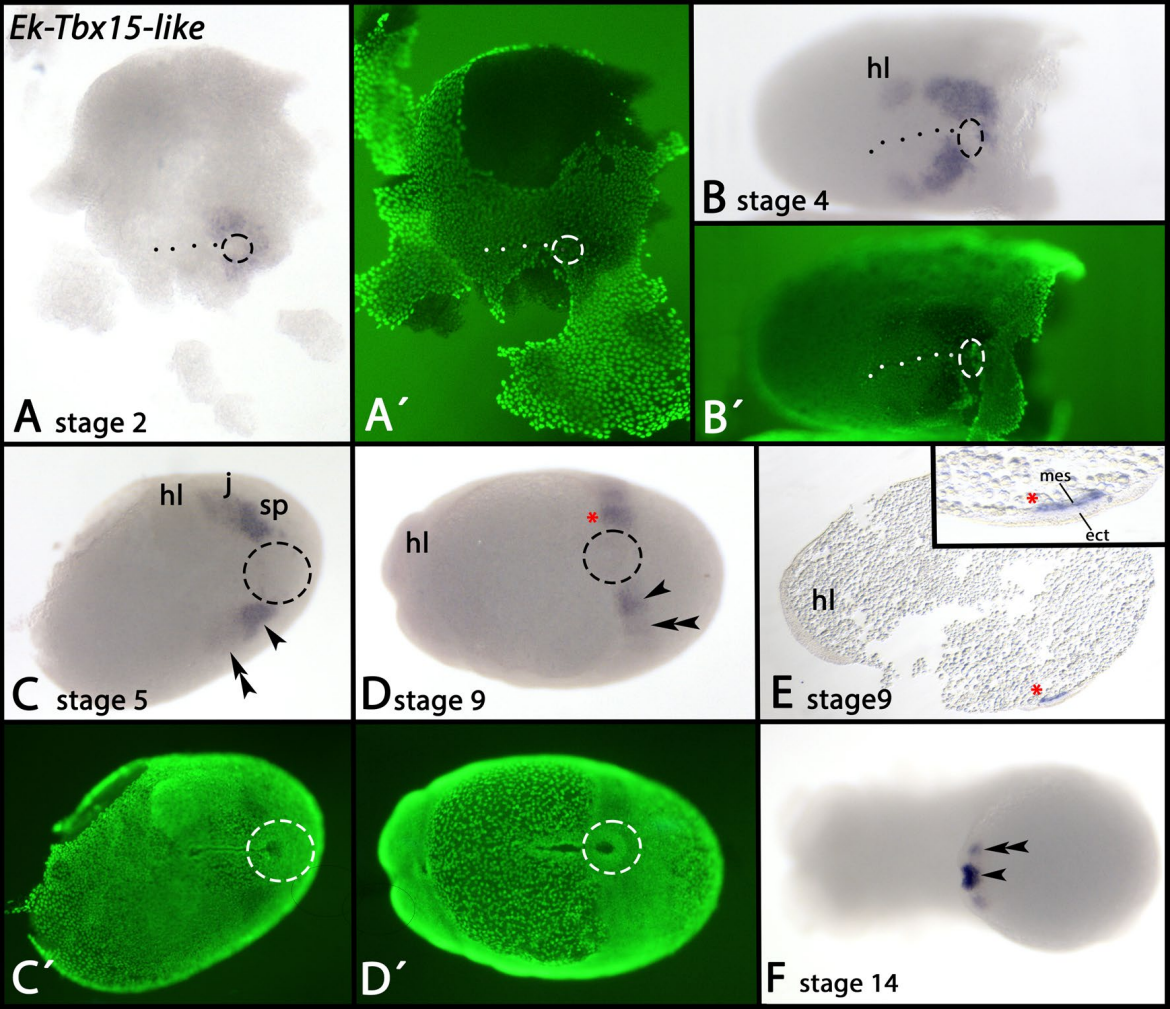
Expression of *Tbx15-like* In all panels, anterior is to the left. Panels A–D and F represent ventral views. Panel E represents a thin section, lateral view, dorsal up. Panels A´–D´ represent SYBR Green staining of the embryos shown in panels A–D. The dashed circles in panels A–D mark the posterior pit region. The dashed line in panels A and B mark the embryonic slit. The red asterisk in panels D and E mark the position in a whole mount (D) and a thin section E. Arrowheads and double arrowheads in panels C, D and F mark expression in the last-formed posterior somites. Developmental stages are indicated (staging system after [9], their supplementary data). Abbreviations: ect, ectoderm; hl, head lobe; j, jaw; mes, mesoderm; sp, slime papilla

*Tbx15-like* is expressed transiently in all developing somites (Fig. 4A–F). This expression is clearly located in the mesoderm as shown by thin sections (Fig. 4E). The posterior pit, however, does not express *Tbx15-like* (Fig. 4A–D, dashed circle). Expression appears with the earliest onset of germ band formation and the development of the first somite (Fig. 4A). After all segments have formed, *Tbx15-like* remains expressed in the posterior of the embryo (Additional file 2: Supplementary Fig. 2C, D, arrowheads).

**Fig. 5:**
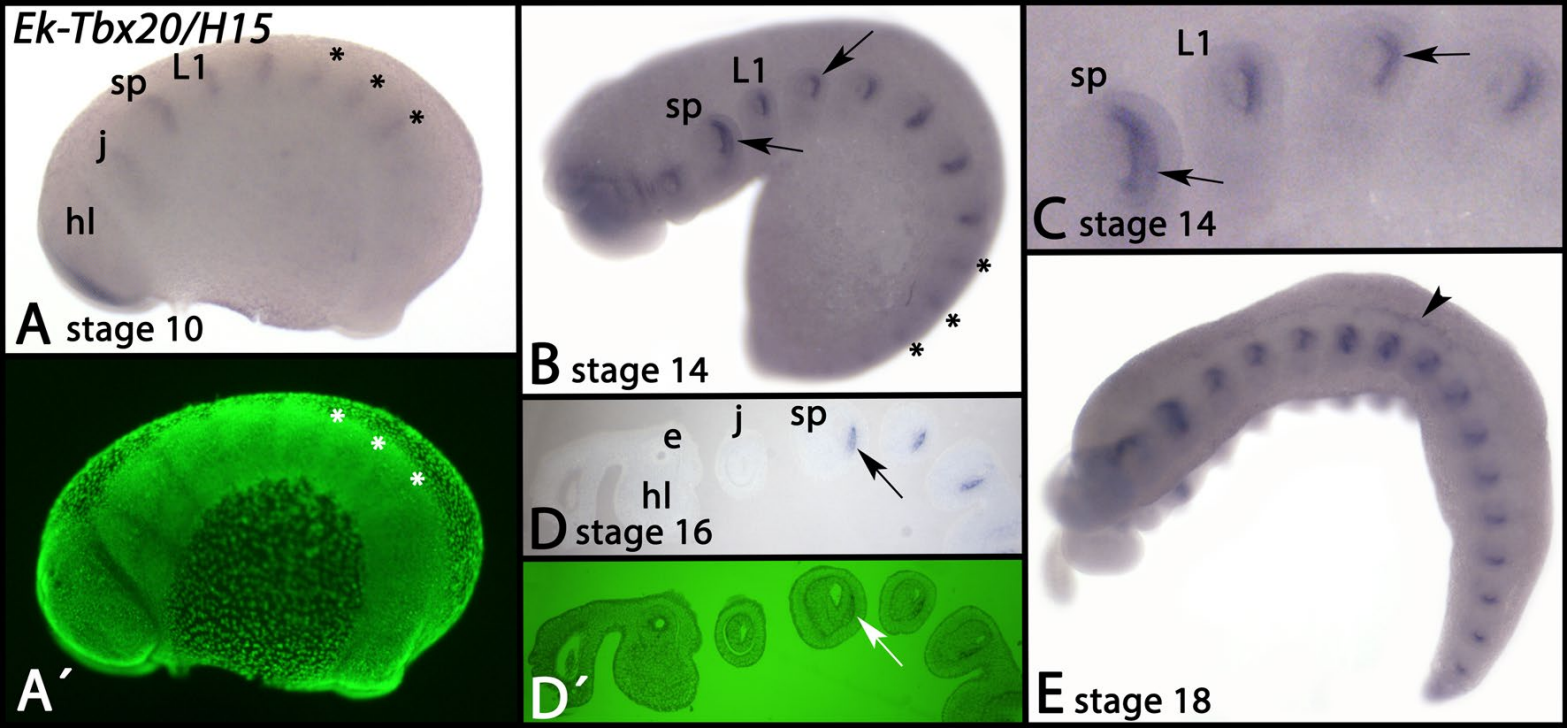
Expression of *Tbx20/H15* In all panels, anterior is to the left, lateral views, dorsal up. Panels A´ and D´ represent SYBR Green staining of the embryos shown in panels A and D. Asterisks in panels A and B mark segmental expression. Arrows in panels B–D point to mesodermal expression in the developing appendages. Panel D represents a thin section. The arrowhead in panel E marks dorsal expression in the developing heart. Developmental stages are indicated (staging system after [9], their supplementary data). Abbreviations: e, eye; hl, head lobe; j, jaw; L, leg; sp, slime papilla

Expression of *Tbx20*/*H15* first appears in the form of transverse segmental stripes at around stage 10 (Fig. 5A, asterisks) (also see [38]. At later developmental stages, segmental expression is located in the posterior of the appendage-mesoderm (Fig. 5B–D, arrows). At around stage 18, expression appears in the developing heart (Fig. 5E, arrowhead) (cf. [10, 38] and [57], the latter for expression of the heart marker gene *tinman*(*tin*)/*NK4* in onychophorans).

**Fig. 6:**
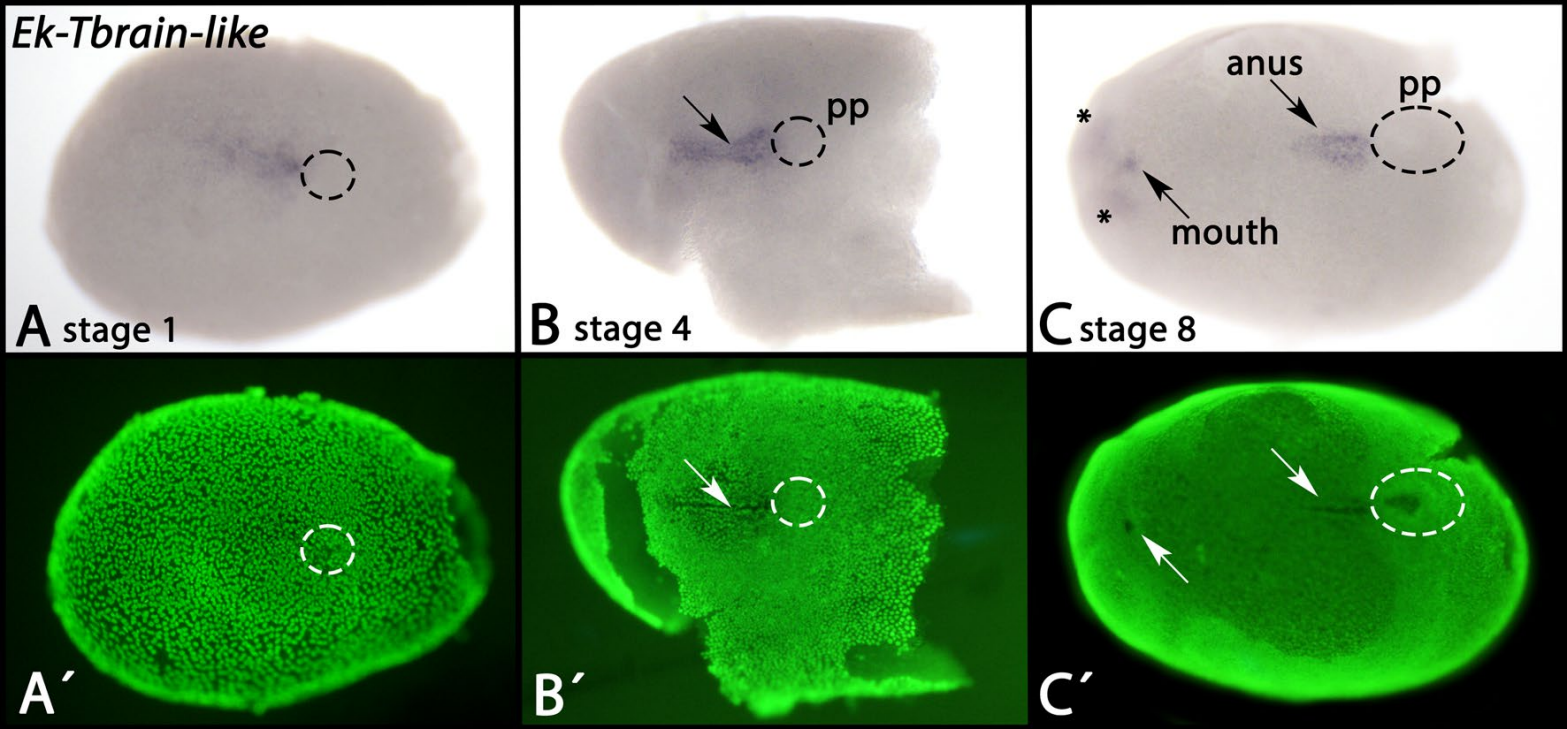
Expression of *Tbrain-like* In all panels, anterior is to the left, ventral views. Panels A´–C´ represent SYBR Green staining of the embryos shown in panels A–C. Arrows point to expression in the embryonic slit. Dashed circles mark the position of the posterior pit. Asterisks in panel C mark expression in the the anterior tissue of the developing embryo that could be associated with the developing brain. Developmental stages are indicated (staging system after [9], their supplementary data). Abbreviations: pp, posterior pit

*Tbrain-like* is expressed prior to the formation of the embryonic slit in the center of the germ disc (Fig. 6A). Later, *Tbrain-like* is expressed in the embryonic slit (Fig. 6B/C, arrows), but not the posteriorly adjacent posterior pit (marked by dashed circles). When the slit closes medially, expression remains in the openings of the future mouth and anus (Fig. 6C). At this point during development, *Tbrain-like* is also expressed in anterior tissue of the embryo that may be associated with the developing brain (Fig. 6C, asterisks) [58] for further information on onychophoran brain development). At later developmental stages, expression in the mouth and the anus disappears, and the aforementioned anterior expression becomes either diffuse or disappears (the detected signal in (Additional file 2: Supplementary Fig. 2E) either represents weak and diffuse expression, or background; note that a similar signal is detectable in the developing appendages).

Expression of the mesodermal genes *twist* (*twi*), *myocyte enhancer factor-2* (*mef2*), *Mesoderm/Mesenchyme homeobox gene* (*Mox*), and *nautilus* (*nau*).

**Fig. 7:**
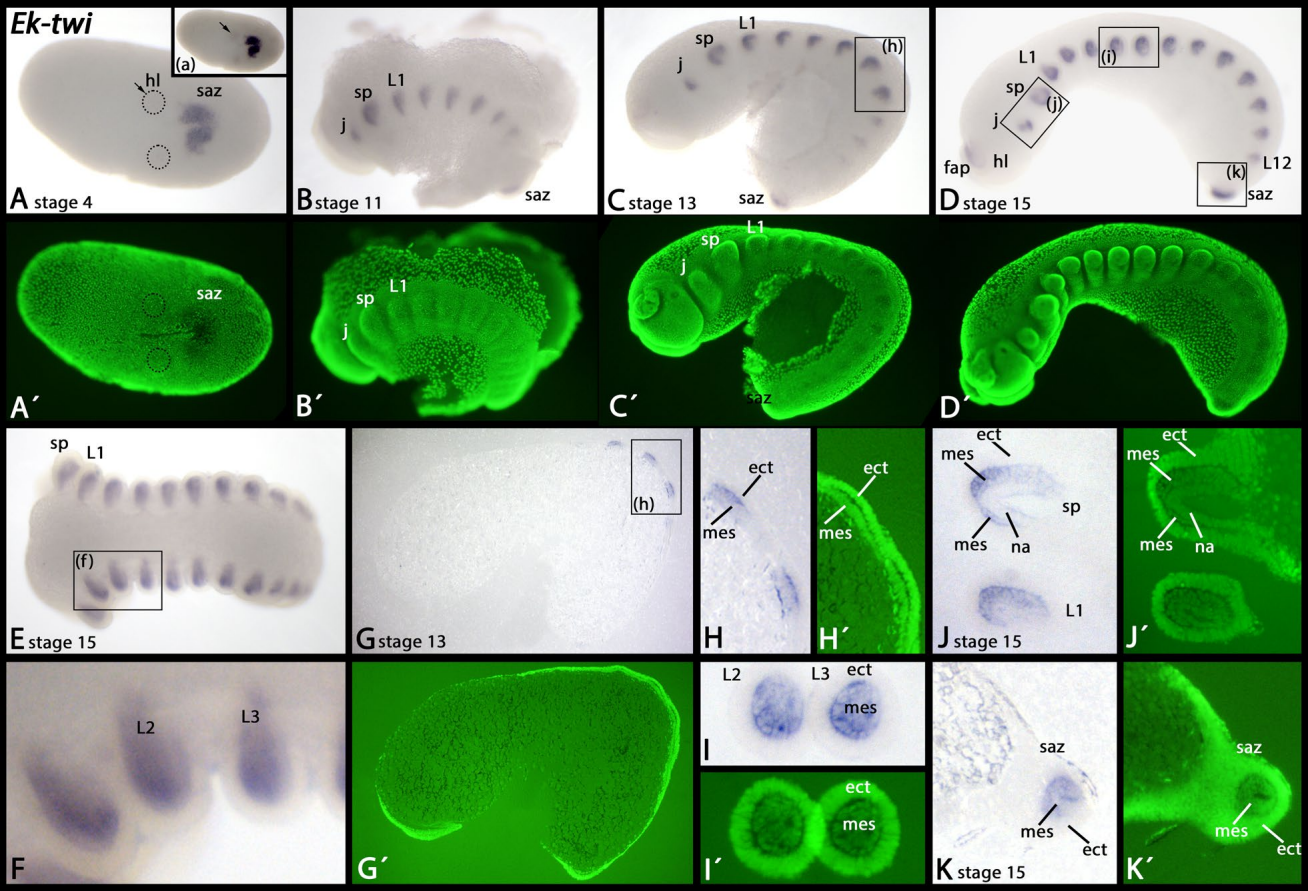
Expression of *twi* In all panels, anterior is to the left, except panel J (anterior up). Panels A represents a ventral view, all other panels represent lateral views (dorsal up). Section (a) (inlay in A) shows the same embryo as in A with increased contrast to better visualize the weak most anterior expression in the somite corresponding to the head lobes (hl) (arrow). The box in panel C marks the same area as shown in panels G and H (thin sections). Panel H shows a magnification of the boxed area in panel G. Boxes in panel D show same areas as in panels I-K (thin sections). Panels A´–D´ and G´–K represent SYBR Green staining of the embryos shown in corresponding panels. Developmental stages are indicated (staging system after [9], their supplementary data). Abbreviations: ect, ectoderm; fap, frontal appendage; hl, head lobe; j, jaw; L, leg; mes, mesoderm; na, nephridial anlagen; saz, segment addition zone; sp, slime papilla

The expression of *Euperipatoides twi* has previously been described for early developmental stages when it is strongly expressed in the posterior pit, and weakly in the most posterior somite [14]. For example at stage 4, the mesoderm of the head lobes has formed and weakly expresses *twi* (Fig. 7A, dashed circles, inlay in A, arrows), while expression in the posterior pit is much stronger (Fig. 7A, inlay in A). At later developmental stages, when the appendages begin developing, *twi* is expressed in the their mesoderm (Fig. 7B–I), except for the mesodermal anlagen of the nephridia (Fig. 7J) (see [59]. This mesodermal expression persists in later developmental stages (Additional file 3: Supplementary Fig. 3D, E). Expression in the posterior pit is also mesodermal (Fig. 7K). Expression of *twi* in the frontal appendages is delayed compared to expression in the other appendages (cf. panels C and E in Fig. 7).

**Fig. 8:**
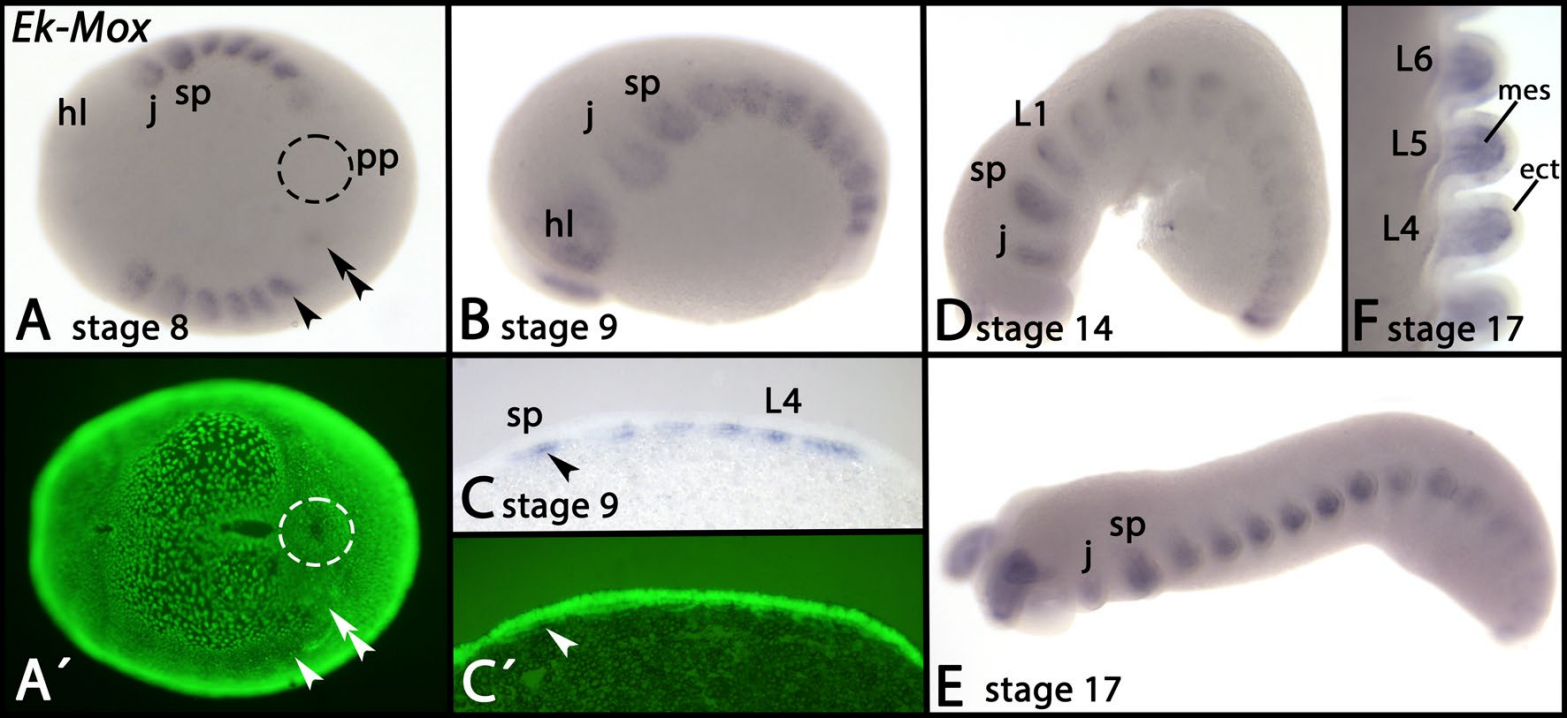
Expression of *Mox* In all panels, anterior is to the left, except panel F (dorsal view, anterior up). Panel A represents a ventral view. Panels B–E represent lateral views, dorsal up Panels A´ and C´ represent SYBR Green staining of the embryos shown in panels A and C. The dashed circle in panel A marks the region of the posterior pit. The arrow and arrowhead in panel A point to expression in the last-formed posterior somites. The arrowhead in panel C (thin section) points to expression in the mesoderm. Developmental stages are indicated (staging system after [9], their supplementary data). Abbreviations: ect, ectoderm; hl, head lobe; j, jaw; L, leg; mes, mesoderm; pp, posterior pit; sp, slime papilla

Expression of *Mox* appears in the somites at around stage 8; note that expression in the most anterior head lobe segment is somewhat delayed compared to the onset of *Mox* expression in more posterior segments (Fig. 8A, B). Cross-sections reveal that this expression is clearly mesodermal (Fig. 8C). At later developmental stages, expression is seen in the entire mesoderm of the appendages, but not the overlying ectoderm (Fig. 8D–F).

**Fig. 9:**
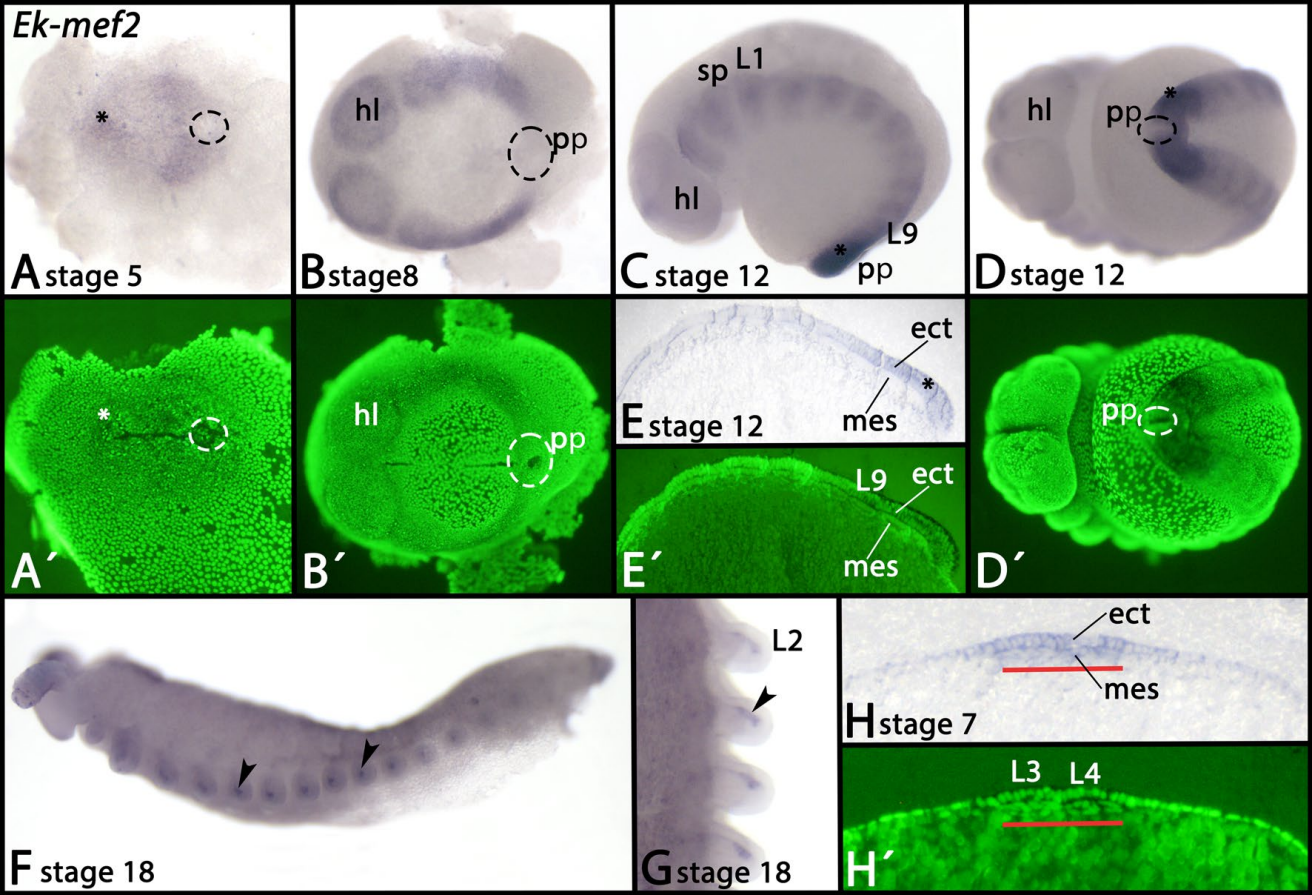
Expression of *mef2* In all panels anterior is to the left, except panel G (anterior up). Panels A, B, D and G represent ventral views. Panels C, E (thin section), F and H (thin section) represent lateral views, dorsal up. Panels A´, B´, D´, E´ and H´ represent SYBR Green staining of the embryos shown in corresponding panels. Dashed circles in panels A, B and D mark the posterior pit region. The asterisk in panel A marks enhanced expression anterior in the embryonic slit. The asterisks in panels C–E mark ectodermal expression overlying the last-formed posterior somite(s). Arrowheads in panels F and G point to expression in the mesoderm of developing appendages. The red bar in panels H/H´ mark the area of mesodermal expression. Developmental stages are indicated (staging system after [9], their supplementary data). Abbreviations: ect, ectoderm; hl, head lobe; L, leg; mes, mesoderm; pp, posterior pit; sp, slime papilla

*Euperipatoides mef2* is strongly expressed in the ectoderm that overlays the posterior somites (Fig. 9A–D), but the posterior pit stays clear of expression (Fig. 9A–D, dashed circles). At later developmental stages, ectodermal expression becomes weak and fully disappears (Fig. 9C–F). At the same time, mesodermal expression appears in an anterior to posterior progression in the somites that later transforms into part of the limb mesoderm (Fig. 9D, F, G, arrowheads). At early developmental stages, ectodermal expression and expression in the underlying somites can co-occur (Fig. 9H).

**Fig. 10:**
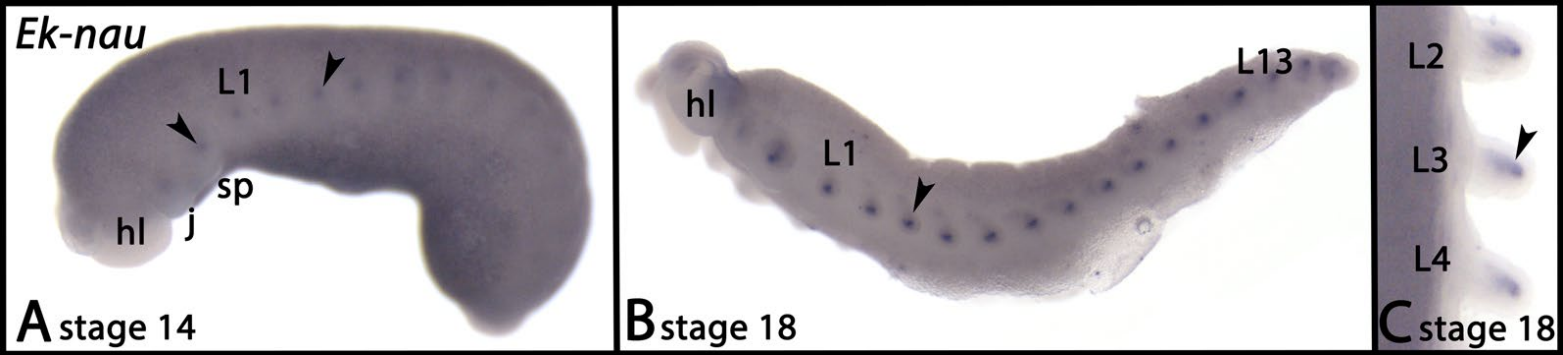
Expression of *nau* In panels A and B anterior is to the left; in panel C anterior is up. Panels A and B represent lateral views, panel C shows as ventral view. The arrowheads point to expression in the mesoderm of developing appendages. Developmental stages are indicated (staging system after [9], their supplementary data). Abbreviations: j, jaw; hl, head lobe; L, leg; sp, slime papilla

Expression of *nau* starts around stage 14 in the anterior part of the mesoderm of the developing appendages (Fig. 10A–C, arrowheads).

### Expression of the endodermal genes *Blimp* and *SoxF*

**Fig. 11:**
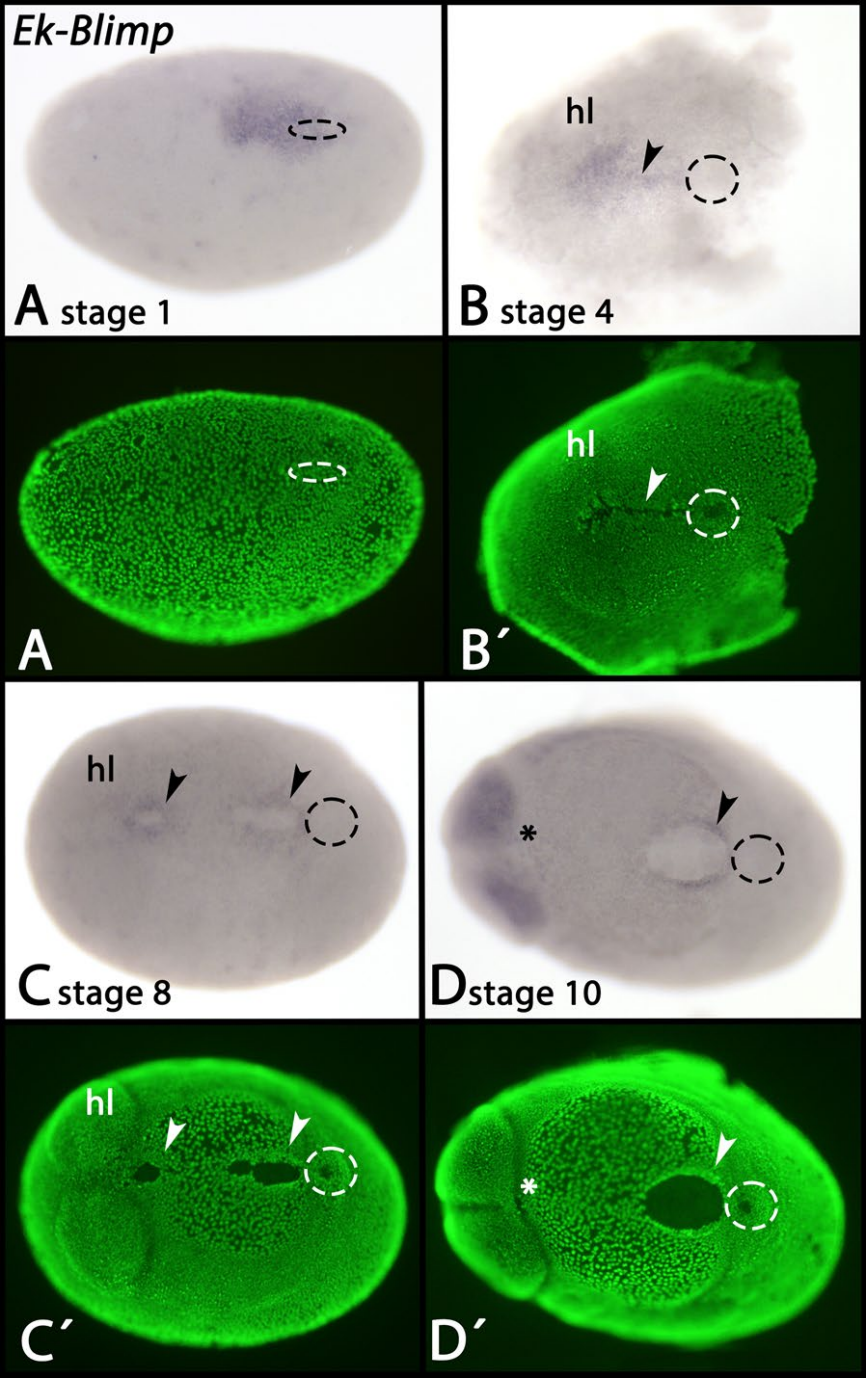
Expression of *Blimp* In all panels, anterior is to the left and ventral views. Panels A´–D´ represent SYBR Green staining of the embryos shown in panels A–D. Dashed lines mark the position of the posterior pit region. Arrowheads point to expression in the embryonic slit. The asterisk in panel D marks the mouth that does not express *Blimp* in later developmental stages. Developmental stages are indicated (staging system after [9], their supplementary data). Abbreviations: hl, head lobe

Before the embryonic slit forms, *Blimp* is already expressed in a broad domain of the blastoderm stage embryo (Fig. 11A). Later, *Blimp* is expressed in the embryonic slit (Fig. 11B–D, arrowheads). Soon after median closure of the slit, however, expression disappears from the future mouth region (Fig. 11D, asterisk), but note that expression in the posterior region of the anus remains (Additional file 2: Supplementary Fig. 2F, arrowhead). At stage 10, additional expression appears in the head lobes (Fig. 11D and Additional file 2: Supplementary Fig. 2F, G). At stage 13, *Blimp* is expressed along the ventral side of the germ band (Additional file 2: Supplementary Fig. 2F) and at later stages, this expression transforms into segmental patches (Additional file 2: Supplementary Fig. 2G).

**Fig. 12:**
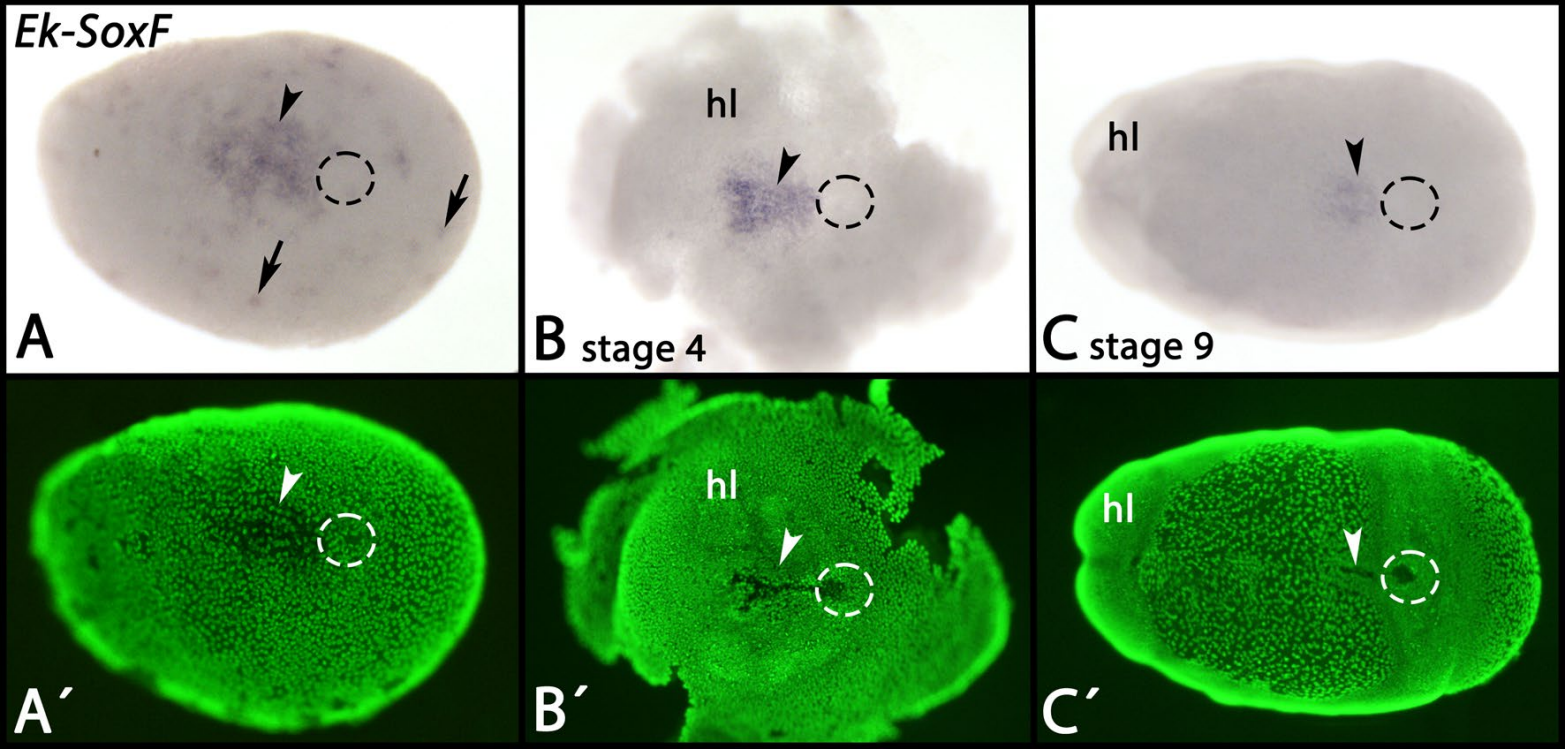
Expression of *SoxF* In all panels, anterior is to the left, ventral views. Panels A´–C´ represent SYBR Green staining of the embryos shown in panels A–C. Dashed circles mark the posterior pit region. Arrows in panel A point to scattered cells that express *SoxF*. Arrowheads point to expression in the embryonic slit. In all panels, the posterior pit is encircled. Developmental stages are indicated (staging system after [9], their supplementary data). Abbreviations: hl, head lobe

At the blastoderm stage, *SoxF* is expressed in form of a large patch anterior the forming posterior pit and before the embryonic slit forms (Fig. 12A, arrowhead). Beyond that, at this stage *SoxF*-expressing cells are also scattered all over the forming germ disc (Fig. 12A, arrows). Later, *SoxF* is expressed in the embryonic slit, but after median closure of the slit, anterior expression disappears (Fig. 12B, C, arrowheads) (also see [48]. Again later during development, expression appears in the dorsal extraembryonic tissue, but unlike other markers of this tissue, *SoxF* is restricted to a central domain within this field of cells, suggesting that the dorsal extraembryonic tissue is not homogenous, but exhibits dorso-ventral differences (Additional file 2: Supplementary Fig. 2H). At stage 13, patches of this dorsal expression remain (Additional file 2: Supplementary Fig. 2I, asterisks) and additional expression appears in the developing nephridia (cf. [48, 59] (Additional file 2: Supplementary Fig. 2I, J).

## Discussion

### Gastrulation, and early endoderm and mesoderm induction

Ancestral functions of the T-box gene *bra* are tightly linked to a positive feedback loop with Wnt-signaling and likely include axis determination, suppression of neuronal genes, and endoderm specification [60, 61], Schwaiger et al. (2020). At least in chordates, *bra* is also important for the induction of the mesoderm [62–65], but it is unclear if this is a generally conserved function in other bilaterian animals (Schwaiger et al. 2020). Another likely ancestral function of *bra* is the regulation of genes involved in morphogenetic movements including the cells of the presumptive early mesoderm and endoderm, and thus gastrulation [4, 65–67]. Typically, therefore *bra* is expressed at the place of gastrulation and the blastoporal lips, but not in the definite endo- and mesoderm. In sea urchins, for example, *bra* is initially expressed in endodermal precursors, but expression in these cells ceases soon after they enter the archenteron [66, 68]. Identified target genes of sea urchin *bra* are either expressed in a similar pattern as *bra*, or in the internalized endodermal cells that then largely do not express *bra* anymore [69]. Similarly, during somite development in vertebrates, *bra* is initially expressed in the primitive streak, but is downregulated in the developing somites [70, 71]. In a basally branching ecdysozoan species, the priapulid worm *Priapulus caudatus*, expression of *bra* in the blastopore during gastrulation suggests a conserved function of this gene [72]. Comparative data on arthropod *bra* (syn. *brachyenteron* (*byn*)) are surprisingly scarce outside *Drosophila* and other insects such as the cricket *Gryllus bimaculatus* and the beetle *Tribolium*. In all of these three insect species, *byn* is involved in hindgut development [73–75]. At least in *Drosophila*, *byn* is also involved in the development of the caudal visceral mesoderm that first expresses *byn* strongly but stops expressing *byn* as the mesoderm moves anteriorly and matures (Kusch and Reuter 1999). A function of *byn* during gastrulation is not reported for insects. In a shrimp, however, *bra* expression is strongest at the gastrula stage suggesting that it may be involved in gastrulation, but whole-mount *in-situ* hybridization data to prove that are not available [76]. Since data on *bry*/*bra* expression in other arthropods are currently not available, we provide supplementary data on *bra* expression in the myriapod *Glomeris* that show that expression at the place of gastrulation and hindgut development are conserved features of *bra, * at least in mandibulate arthropods (Additional file 4: Supplementary Fig. 4A–D).

Another class of T-box genes that is involved in endoderm and mesoderm development is *Tbx6*, a target of *bra* reviewed i [22, 77], that diverged into *Tbx6*, *vegT* and *MGA* in vertebrates (with *vegT* being lost in mammals) [53]. One key function of *Tbx6*-class genes in vertebrates is the determination of the endomesoderm, another is organization, separation and development of endoderm and mesoderm [78–86], (reviewed in Wardle and Papaioannou [22]). Like *bra*, *Tbx6-class* genes are expressed in the primitive streak in vertebrates, but are not so in the forming mesodermal somites [71, 87, 88]. Unfortunately, comparative data on *Tbx6*-class genes in panarthropods are restricted to the insects *Drosophila* and *Tribolium* where the *Tbx6* orthologs (*dorsocross* (*doc*) genes) are not involved in gastrulation and early mesoderm and endoderm induction (reviewed in Horn and Panfilio [21, 89]).

A third player in this concert appears to be *Wnt11*. At least in vertebrates, *Wnt11* is involved in mesoderm induction, endoderm induction, convergent extension during gastrulation, axis elongation and archenteron extension [90–94]. Data on *Wnt11* function outside the vertebrates, however, is scarce, and even expression and presence in the genome is by no means conserved in all hitherto studied species. Although *Wnt11* has been lost in the lineage leading to *Drosophila* and the lack of early and posterior expression in *Tribolium* has been reported [95], at least in myriapods and some chelicerates, *Wnt11* is expressed early and continuously in the posterior of the embryo, the putative site of gastrulation [96–98]. The loss of *Wnt11* in some groups of animals and the lack of *Wnt11* expression during gastrulation in others may be best explained with function shuffling as reported previously for Wnt-class genes [99], Janssen et al. 2021, [100]. Of interest for this study, however, is the fact that in the onychophoran *Euperipatoides*, *Wnt11* is expressed continuously in the posterior pit in a very similar pattern as *bra* and *Tbx6-like* [101].

The expression patterns of onychophoran *bra* [10], *Tbx6-like* (Fig. 3), and *Wnt11* [101] thus are comparable with their orthologs during gastrulation in most other animals: all three genes are expressed early during onychophoran development in the posterior pit, likely in endomesodermal precursors, but are not expressed in definitive mesodermal and endodermal cells as their expression is restricted to the posterior pit region.

### Conserved aspects of endoderm development

We previously studied the early expression patterns of the conserved bilaterian endoderm marker genes *Hepatocyte nuclear factor 4* (*Hnf4*), *GATA456*, and *forkhead* (*fkh*) in the onychophoran, and found that these genes all are expressed early during development in the embryonic slit suggesting that these cells represent the earliest definite endoderm [10, 18].

The early expression of the newly-discovered onychophoran *Tbrain-like* gene is also restricted to the embryonic slit (Fig. 6). This expression appears to be comparable with the expression of *Tbrain* in other animals during gastrulation and endoderm development: in vertebrates, *Tbrain* is expressed *inter alia* in the early developing endoderm (reviewed in Probst and Arnold [102]), in the hemichordate *Ptychodera flava*, *Tbrain* is expressed at the base of the invaginating archenteron [103], and in the cephalochordate amphioxus and echinoderms, *Tbrain* is expressed in the archenteron during the process of gastrulation [104–109]. Early expression and function during gastrulation is thus conserved in deuterostomes, but also in a lophotrochozoan/spiralian animal, the polychaete *Hydroides elegans*, *Tbrain* is expressed in endodermal precursors that originate from the blastoporal region [34]. Therefore, *Tbrain/Tbrain-like* appears to represent another conserved marker of the early endoderm of bilaterian animals including onychophorans.

In bilaterian animals including chordates, echinoderms, and polychaetes*, Blimp* is expressed in the developing early endoderm (e.g. [28, 32, 110, 34]). In the polychaete *Hydroides elegans*, for example, *Blimp* is expressed in the invaginated cells of the archenteron, but not the blastopore lips [34]. Similarly, in the sea urchin *Strongylocentrotus purpuratus* and the starfish *Asterina miniata*, *Blimp* is expressed in the invaginated cells of the archenteron [32, 111]. In insects such as *Drosophila* and the cricket *Gryllus bimaculatus*, however, *Blimp* does not seem to play a role in early endoderm development [112, 113]. In *Drosophila*, expression suggests a role in the development of the precursors of the peripheral nervous system, the tracheal system, and the developing hindgut [112]. Since data from other arthropods were not available prior to this study, we investigated the expression of *Blimp* in the myriapod *Glomeris* and the spider *Parasteatoda*. The expression of these genes suggest a conserved role of *Blimp* in nervous system development in arthropods, but indeed does not suggest involvement in early endoderm development (Fig. 1C and Additional file 4: Supplementary Fig. 4E-P). The observed expression pattern of *Euperipatoides blimp*, however, suggest that the function in nervous system development evolved in the lineage leading to Arthropoda and that the ancestral function as endoderm-patterning gene is retained in onychophorans (Fig. 11).

Expression of *Euperipatoides SoxF* has previously been described for mid-to-late embryonic stages when it is expressed in the posterior of the embryonic slit [48]. Because *SoxF*-type Sox genes are involved in endoderm development in vertebrates and are direct targets of *Tbx6* [27, 29, 114, 115], we re-investigate the expression of *SoxF* in the earliest accessible developmental stages of the onychophoran. In these stages, *SoxF* is indeed strongly expressed in the lips of the embryonic slit and the surrounding ventral extra-embryonic tissue. This suggests that *SoxF* may act as an early target of *Tbx6* genes in vertebrates and onychophorans. If this is a result of deep conservation of the endoderm-patterning network or a result of convergence is unclear. In arthropods, however, a function of *SoxF* in endoderm development likely is not conserved [48, 116].

### Conserved aspects of mesoderm development

In onychophorans, *twist* (*twi*), a conserved regulator of mesoderm development [117–122], is expressed in the mesoderm underlying the posterior pit (Fig. 7) [14]. *twi* is thus expressed in the earliest formed mesoderm underlying the posterior ectoderm that expresses *bra* and *Tbx6-like*. This is comparable with the situation in other animals, were *bra* and *Tbx6/doc* regulate mesodermal gene expression, but are not expressed in the definitive mesoderm. The expression of onychophoran *twi*, however, is in line with a conserved function in early mesoderm formation under control of *bra* and/or *Tbx6-like*.

**Fig. 13:**
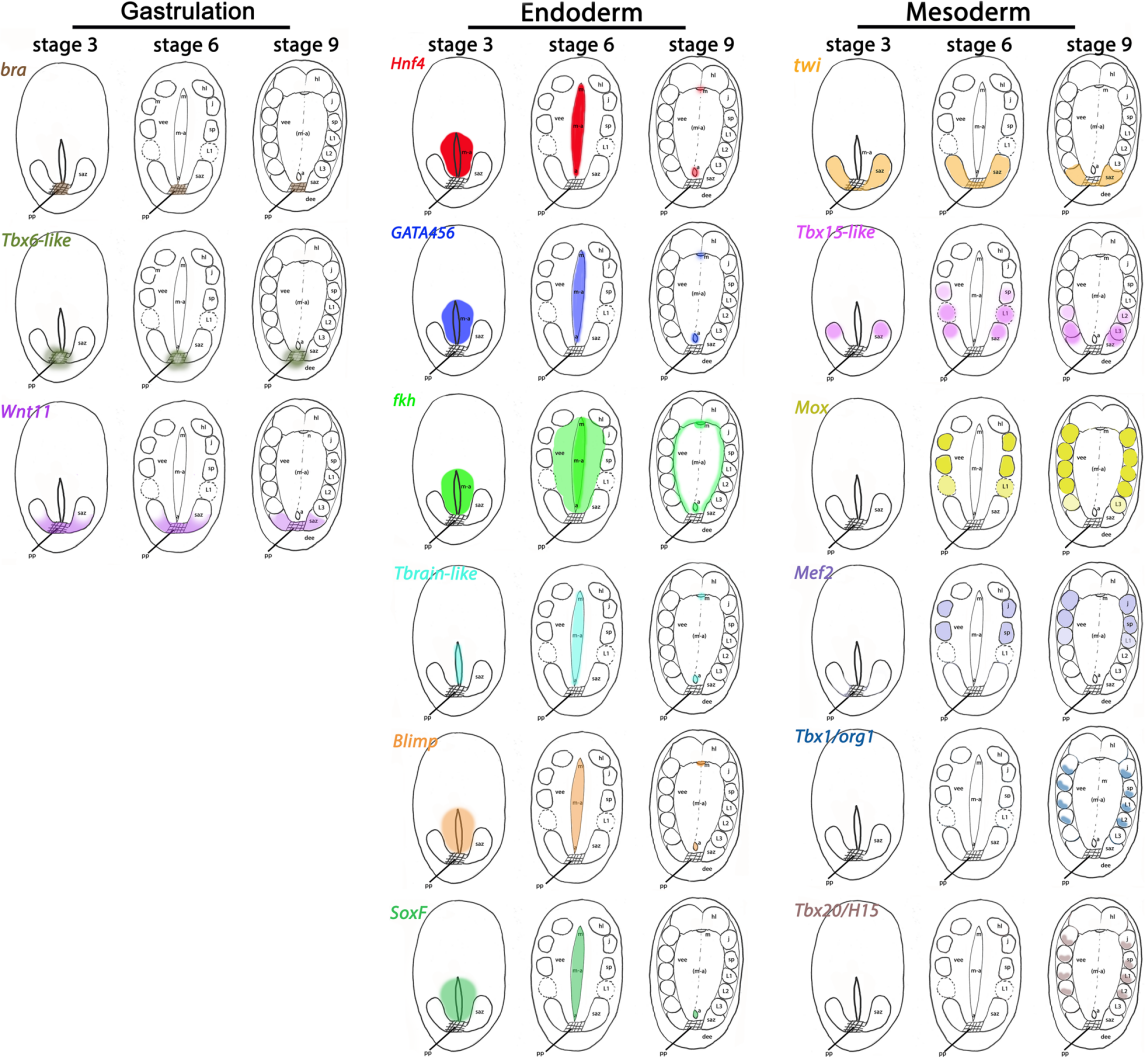
Schematic overview, expression of endoderm- and mesoderm-patterning genes The early expression of marker genes is shown in gene-specific colours. In the schematic drawings of embryos, anterior is pointing upwards, ventral views. Abbreviations: a, anus; dee, dorsal extraembryonic tissue; hl, somite of the head lobe segment; j, somite of the jaw-bearing segment; L, somite of a leg-bearing segment; m, mouth; m-a, mouth-anus furrow (the embryonic slit); pp, posterior pit (blastopore); saz, segment addition zone; sp, somite of the slime papilla-bearing segment; vee, ventral extraembryonic tissue

Another potential factor of mesoderm development is *Tbx15*, a member of the *Tbx1/15/20*-class of T-box genes [123–126]. *Tbx15* evolved in early metazoan lineages as it is present in sponges and ctenophores [53]. In the latter, the single *Tbx1/15/20* ortholog is expressed in the developing mesendodermal tissue and the edges of the blastopore [126]. In the stem leading to Cnidaria + Bilateria, this gene duplicated into three separate classes, *Tbx1*, *Tbx15* and *Tbx20*. While *Tbx1* and *Tbx20* have been retained in all lineages of Bilateria, *Tbx15* has been lost from some bilaterian lineages (reviewed in Sebé-Pedrós and Ruiz-Trillo [53]). Interestingly, the onychophoran *Tbx15-like* gene is expressed prominently in the newly forming somites on either side of the posterior pit, and thus resembling the expression of the mesodermal marker *twi*, although expression of *Tbx15-like* in the developing somites is delayed compared to *twi* [14] (Figs. 4 and 13). Expression of onychophoran *Tbx15-like* thus is comparable with its expression in other species that retained *Tbx15* such as ctenophores, cephalochordates and vertebrates [123, 124, 126] suggesting that the function of *Tbx15* genes in early mesoderm development is conserved throughout metazoan evolution.

*Mox* is a conserved factor of mesoderm development and possibly also myogenesis in bilaterian animals (reviewed in Schulreich et al. [127]). In vertebrates and cephalochordates, *Mox* genes are transiently expressed in the forming somites early during mesoderm development [24, 128], and in lophotrochozoans/spiralians, *Mox* is equally early expressed in the developing paired mesodermal bands Passamaneck et al. (2015), [129], Sun et al. 2022). Information on Mox expression and function in ecdysozoans is scarce. The *Drosophila* ortholog, *buttonless* (*btn*), is involved in mesoderm development [42], albeit at later developmental stages in the developing dorsal median (DM) cells that are of mesodermal origin [130, 131]. We show that this function of *Mox*/*btn* likely dates back to the last common ancestor of arthropods as revealed by the conserved pattern of *Mox* in the myriapod *Glomeris* and the spider *Parasteatoda* (Additional file 4: Supplementary Fig. 4Q-W). The onychophoran data thus provide the first evidence for a conserved role of *Mox* in early mesoderm development in any ecdysozoan species. This suggests a change of *Mox* function from early mesoderm development to a function in the development of the DM cells in the lineage leading to Arthropoda. The reason for this, however, remains unclear.

In *Drosophila*, *mef2* first is expressed in all mesoderm under the control of *twi*, but later is restricted to certain subtypes of differentiating mesodermal tissue [45, 132, 133]. In the crustacean *Parhyale hawaiensis*, *twi* and *mef2* are not activated prior to the proliferation of segmental mesoderm, and hence comparably later than in *Drosophila*, the regulatory interaction of *twi* and *mef2*, however, may be conserved [134]. Similarly, in spiders, *mef2* appears relatively late during development in mesodermal cells including the developing heart [135, 136]. At least in *Drosophila* and *Parhyale*, *mef2* expression is not limited to mesodermal/muscle cells, but is also expressed in ectodermal derivatives such as the developing nervous system [134, 137]. In *Glomeris*, however, *mef2* is expressed early during development in the ectoderm of newly forming segments and the foregut primordium, and later also in the dorsal mesoderm and the mesoderm of the anal valves (Additional file 4: Supplementary Fig. 4X-c). The early ectodermal expression of *mef2* in early forming segments of the onychophoran is thus only shared with the myriapod. The later expression in the developing and differentiating somites and the musculature of the appendages, however, likely represent conserved features of *mef2* in mesoderm development (Fig. 9).

In bilaterian animals including the fly *Drosophila*, *Tbx1* (*org1*) and *Tbx20* (*H15*) genes are expressed *inter alia* in mesodermal derivatives such as certain types of muscles, gut parenchyme, the heart, and developing somites [21, 138–144]. In onychophorans, *Tbx1/org1* and *Tbx20/H15* both are expressed in mesodermal tissue of newly formed (posterior) segments, and later also in part of the mesoderm of the developing appendages, and thus their expression is in line with a function in mesoderm development and specification as it is the case in other bilaterian animals (Figs. 2 and 5) [38].

The myogenic gene *nau/MyoD* generally is involved in mesoderm differentiation and muscle development in bilaterian animals [145–148]. In the brachiopod *Terebratalia transversa* for example, *MyoD* first is expressed at gastrulation and early during mesoderm development, but later also persists being expressed during myogenic specification and mesoderm differentiation [149]. Similarly, in the sea urchin *Lytechinus variegatus*, *MyoD* (*Sum1*) is expressed in mesodermal cells during gastrulation prior to myocyte differentiation [150]. In *Drosophila*, *nau* is first expressed in mesodermal cells just prior to the differentiation of mesoderm into muscle precursors and the fusion of muscle cells (Michelson et al. [23]. Likewise, in the myriapod *Glomeris*, *nau* is expressed relatively late during development in the prominent dorsal musculature of this animal [151]. The late onset of *nau* expression in the onychophoran is thus in line with a conserved function of this gene in muscle differentiation.

From the available published data, and the expression patterns presented in this study, we conclude that in onychophorans, *twi* is an early (or the earliest) marker of definitive mesodermal tissue, possibly under direct or indirect control of *bra* and/or *Tbx6-like*. The temporally staggered expression patterns of *twi*, *Tbx15-like* and *Mox* in the developing somites (Fig. 13) suggests consecutive functions in somite maturation and thus mesoderm differentiation. The expression of *mef2*, *Tbx1/org1*, *Tbx20/H15*, and *nau* which all are expressed in the developing mesoderm long after the onset of *twi*, *Tbx15-like* and *Mox* expression suggest a function in later mesoderm and muscle differentiation (summarized in Fig. 13).

### The onychophoran blastopore

The conserved expression patterns reported in this paper are in line with our previous suggestions that endoderm is induced or originates from the anterior rim of the posterior pit, that mesoderm is induced or originates from the remaining part of the posterior pit, and that the posterior pit therefore represents the onychophoran blastopore [5, 5, 10] showed that endoderm forms already prior to the development of the embryonic slit. This corroborates with our earlier findings that endodermal markers such as *Hnf4* are expressed in cells anterior to the blastopore (sensu Manton, Kennel, Janssen) *before* the embryonic slit forms [18]. And indeed, the new data on the early endoderm marker genes *Tbrain-like*, *SoxF* and *Blimp* also corroborate these findings as all three genes are expressed prior to the development of a slit. It is thus very unlikely that endoderm originates from the lips of the slit, although this may indeed be the place from which a fraction of endodermal cells sink into the yolk as previously described for *Euperipatoides* (Eriksson and Tait [8]. In addition, the persisting expression of *bra*, *Tbx6-like* and indeed also the endoderm markers *forkhead* (*fkh*) [10], *Hnf4* [18] (Additional file 3: Supplementary Fig. 3A-C), *GATA456* [18] and *SoxF* [48] anterior adjacent to the posterior pit and *after* closure of the embryonic slit suggests that endodermal cells still originate at later developmental stages from this region and in the absence of an embryonic slit, as previously suggested by other authors [12, 13, 17]. Likewise, it appears that mesodermal cells still originate from the posterior pit at comparably late developmental stages as suggested by the persisting expression of the early mesodermal markers *twi* [14] (Additional file 3: Supplementary Fig. 3D, E) and *Tbx15-like* (Fig. 4).

In summary, these data suggest that the posterior pit of onychophorans represents the blastopore (blastoporal lips) and that the embryonic slit (the mouth-anus furrow) represents a unique structure of onychophoran development, likely as an adaptation to the high degree of yolk seen in most groups of onychophorans [5]. Indeed, an embryonic slit does not form in yolkless placental onychophorans. Their gastrulation has been described as invagination of cells and thus formation of endoderm and mesoderm from a blastopore at the vegetal pole [17], (reviewed in Mayer et al. [6]), a process that is similar to that described for other groups of bilaterian animals. The situation in onychophorans with yolky eggs is thus likely derived. Manton speculated that “*When the increase in the yolk led to the formation of a single flat disk of blastomeres lying upon the yolk, the original vegetal pole cells would lie at the periphery of the disk, …*” [5]. We suggest a similar but different scenario in which the posterior pit indeed represents the blastopore and that the field of cells that expresses early endodermal marker genes anterior to the posterior pit represents an archenteron-like structure that in yolkless onychophorans is internalized (as seen in many other groups of bilaterian animals), and that is located on the surface of the embryo in yolk-rich onychophorans such as *Euperipatoides*.

This hypothesis is testable via the investigation of spatiotemporal patterns of endodermal and mesodermal marker gene expression in the yolkless embryos of placental onychophorans. If our suggestion is true, the same endodermal markers as investigated in *Euperipatoides* will be expressed in internalized tissues of the gastrula in yolkless placental onychophoran embryos, and early mesodermal and endoderm/mesoderm-inducing genes will be expressed at the rim and the center of the blastopore in such embryos.

### Supplementary Information

**Additional file1**: Phylogenetic tree of arthropod T-box genes. Bayesian analyses. The scale bar represents the amino acid substitutions rate per site. Species and accession numbers are listed in Supplementary File 7. See text for further information.

**Additional file 2**: Additional aspects of onychophoran gene expression. Expression of Tbx6-like (A, B), Tbx15-like (C, D), Tbrain-like (E), Blimp (F, G) and SoxF (H-J). In all panels, anterior is to the left. Panels A, C, E, and F represent lateral views, anterior up. Panels B, H, I and J represent dorsal views. Panels D and G represent ventral views. Panel H´ represents a SYBR Green staining of the embryo shown in panel H. Asterisks in panels A, B and I mark expression/signal in the dorsal extraembryonic tissue. Double arrowheads in panels A and B point to expression dorsal in the appendages. The arrowheads in panels A, C, D and F point to expression in the posterior pit region. Note that SoxF expression is only expressed in the centre of the extraembryonic tissue (the dorsal field, df); the dorsal-ventral extent of the dorsal field is marked by red bars in panels H/H´. Filled circles in panel J mark potential segmental endodermal expression. Abbreviations as in Figure 2; df, dorsal field.

**Additional file 3**: Late Expression of Hnf4 and twi. In all panels, anterior is to the left, ventral views (except panel C that shows a lateral view). Arrows point to posterior expression associated with de novo formation of endoderm and mesoderm. Abbreviations as in Figure 2.

**Additional file 4**: Arthropod gene expression. Expression of Glomeris marginata bra (A-D), Blimp (E-G), Mox (Q-S), and mef2 (X-c). Expression of Parasteatoda tepidariorum BlimpA (H-K), BlimpB (L-P), and Mox (T-W). In all panels, anterior is to the left and ventral views, except panels H and W that represent lateral50views. Panels indicated with an apostrophe (´) show SYBR Green staining of corresponding embryos shown in the bright field photographs. The arrow in panel B points to expression in the developing hindgut, the arrow points to expression in the developing anal valves. Arrows in panels Q-W point to expression in the developing central nervous system on either side of the ventral midline. Arrowheads in panels a-c mark expression in the developing musculature associated with the dorsal segmental units of the trunk. Abbreviations: aSP, anterior spinneret; av, anal valves; ch, chelicera; hg, hindgut; hl, head lobe; L, leg; md, mandible; oc, ocular region; s, stomodaeum; saz, segment addition zone; T, trunk segment.

**Additional file 5**: Tbox Nexus File.

**Additional file 6**: Tbox Nexus File (arthropod tree).

**Additional file 7**: Gene identifiers and primers.

**Additional file 8**: Mox Nexus File.

**Additional file 9**: Blimp Nexus File.

## Data Availability

All data in this study are provided as supplementary materials.

## References

[1] Wu R, Pisani D, Donoghue PCJ (2023). The unbearable uncertainty of panarthropod relationships. Biol Lett.

[2] Nájera GS, Weijer CJ (2023). The evolution of gastrulation morphologies. Development.

[3] Budd GE, Jensen S (2000). A critical reappraisal of the fossil record of the bilaterian phyla. Biol Rev.

[4] Technau U, Scholz CB (2003). Origin and evolution of endoderm and mesoderm. Int J Dev Biol.

[5] Manton SM (1949). Studies on the Onychophora VII. The early embryonic stages of Peripatopsis, and some general considerations concerning the morphology and phylogeny of the arthropoda. Philos Trans R Soc B-Biol Sci.

[6] Mayer G, Franke FA, Treffkorn S, Gross V, Oliveira IS, Wanninger A (2015). Onychophora. Evolutionary Developmental Biology of Invertebrates 3: Ecdysozoa I: Non-Tetraconata.

[7] Treffkorn S, Mayer G, Janssen R (2022). Review of extra-embryonic tissues in the closest arthropod relatives, onychophorans and tardigrades. Philos Trans R Soc Lond B Biol Sci.

[8] Eriksson BJ, Tait NN (2012). Early development in the velvet worm Euperipatoides kanangrensis Reid 1996 (Onychophora: Peripatopsidae). Arthropod Struct Dev.

[9] Janssen R, Budd GE (2013). Deciphering the onychophoran 'segmentation gene cascade': Gene expression reveals limited involvement of pair rule gene orthologs in segmentation, but a highly conserved segment polarity gene network. Dev Biol.

[10] Janssen R, Jörgensen M, Lagebro L, Budd GE (2015). Fate and nature of the onychophoran mouth-anus furrow and its contribution to the blastopore. Proc Biol Sci.

[11] Sedgwick A (1885). The development of Peripatus capensis. Proc R Soc Lond B Biol Sci.

[12] Anderson DT (1966). The comparative early embryology of the Oligochaeta, Hirudinea and Onychophora. Proc Linnean Soc NSW.

[13] Evans R (1901). On the Malayan species of Onychophora. Part II. The development of Eoperipatus weldoni. Quarterly Journal of Microscopical Science.

[14] Janssen R (2017). A molecular view of onychophoran segmentation. Arthropod Struct Dev.

[15] Balfour FM (1883). The anatomy and development of Peripatus capensis. Q J Microsc Sci.

[16] Sheldon L (1888). On the development of *Peripatus novae-zealandiae*. Q J Microsc Sci.

[17] Kennel J (1885). Entwicklungsgeschichte von Peripatus edwardsii Blanch. und Peripatus torquatus n.sp. I. Theil Arb Zool Zootom Inst Wrzburg.

[18] Janssen R, Budd GE (2017). Investigation of endoderm marker-genes during gastrulation and gut-development in the velvet worm Euperipatoides kanangrensis. Dev Biol.

[19] Nielsen C, Brunet T, Arendt D (2018). Evolution of the bilaterian mouth and anus. Nat Ecol Evol.

[20] Showell C, Binder O, Conlon FL (2004). T-box genes in early embryogenesis. Dev Dyn.

[21] Reim I, Frasch M, Schaub C (2017). T-Box Genes in Drosophila Mesoderm Development. Curr Top Dev Biol.

[22] Wardle FC, Papaioannou VE (2008). Teasing out T-box targets in early mesoderm. Curr Opin Genet Dev.

[23] Michelson AM, Abmayr SM, Bate M, Arias AM, Maniatis T (1990). Expression of a MyoD family member prefigures muscle pattern in Drosophila embryos. Genes Dev.

[24] Candia AF, Hu J, Crosby J, Lalley PA, Noden D, Nadeau JH, Wright CV (1992). Mox-1 and Mox-2 define a novel homeobox gene subfamily and are differentially expressed during early mesodermal patterning in mouse embryos. Development.

[25] Olson EN, Perry M, Schulz RA (1995). Regulation of muscle differentiation by the MEF2 family of MADS box transcription factors. Dev Biol.

[26] Wang W, Wikramanayake AH, Gonzalez-Rimbau M, Vlahou A, Flytzanis CN, Klein WH (1996). Very early and transient vegetal-plate expression of SpKrox1, a Krüppel/Krox gene from Stronglyocentrotus purpuratus. Mech Dev.

[27] Hudson C, Clements D, Friday RV, Stott D, Woodland HR (1997). Xsox17alpha and -beta mediate endoderm formation in Xenopus. Cell.

[28] de Souza FS, Gawantka V, Gómez AP, Delius H, Ang SL, Niehrs C (1999). The zinc finger gene Xblimp1 controls anterior endomesodermal cell fate in Spemann's organizer. EMBO J.

[29] Clements D, Woodland HR (2000). Changes in embryonic cell fate produced by expression of an endodermal transcription factor, Xsox17. Mech Dev.

[30] Hinman VF, Degnan BM (2002). Mox homeobox expression in muscle lineage of the gastropod Haliotis asinina: evidence for a conserved role in bilaterian myogenesis. Dev Genes Evol.

[31] Davidson EH, Rast JP, Oliveri P, Ransick A, Calestani C, Yuh CH, Minokawa T, Amore G, Hinman V, Arenas-Mena C, Otim O, Brown CT, Livi CB, Lee PY, Revilla R, Schilstra MJ, Clarke PJ, Rust AG, Pan Z, Arnone MI, Rowen L, Cameron RA, McClay DR, Hood L, Bolouri H (2002). A provisional regulatory gene network for specification of endomesoderm in the sea urchin embryo. Dev Biol.

[32] Hinman VF, Davidson EH (2003). Expression of Am Krox, a starfish ortholog of a sea urchin transcription factor essential for endomesodermal specification. Gene Expr Patterns.

[33] Wei Q, Rong Y, Paterson BM (2007). Stereotypic founder cell patterning and embryonic muscle formation in Drosophila require nautilus (MyoD) gene function. Proc Natl Acad Sci U S A.

[34] Arenas-Mena C (2008). The transcription factors HeBlimp and HeT-brain of an indirectly developing polychaete suggest ancestral endodermal, gastrulation, and sensory cell-type specification roles. J Exp Zool B Mol Dev Evol.

[35] Boyle MJ, Yamaguchi E, Seaver EC. Molecular conservation of metazoan gut formation: evidence from expression of endomesoderm genes in *Capitella teleta* (Annelida). Evodevo. 2014;5:39. 10.1186/2041-9139-5-39. 10.1186/2041-9139-5-39 PMC4407770 25908956

[36] Sachslehner A, Zieger E, Calcino A, Wanninger A (2021). HES and Mox genes are expressed during early mesoderm formation in a mollusk with putative ancestral features. Sci Rep.

[37] Junion G, Jagla K (2022). Diversification of muscle types in Drosophila embryos. Exp Cell Res.

[38] Janssen R, Jörgensen M, Prpic NM, Budd GE (2015). Aspects of dorso-ventral and proximo-distal limb patterning in onychophorans. Evol Dev.

[39] Huelsenbeck JP, Ronquist F (2001). MRBAYES: Bayesian inference of phylogenetic trees. Bioinformatics.

[40] Panara V, Budd GE, Janssen R (2019). Phylogenetic analysis and embryonic expression of panarthropod Dmrt genes. Front Zool.

[41] Minguillón C, Garcia-Fernàndez J (2003). Genesis and evolution of the Evx and Mox genes and the extended Hox and ParaHox gene clusters. Genome Biol.

[42] Chiang C, Patel NH, Young KE, Beachy PA (1994). The novel homeodomain gene buttonless specifies differentiation and axonal guidance functions of Drosophila dorsal median cells. Development.

[43] Janssen R, Posnien N (2014). Identification and embryonic expression of Wnt2, Wnt4, Wnt5 and Wnt9 in the millipede Glomeris marginata (Myriapoda: Diplopoda). Gene Expr Patterns.

[44] Schwager EE, Sharma PP, Clarke T, Leite DJ (2017). The house spider genome reveals an ancient whole-genome duplication during arachnid evolution. BMC Biol.

[45] Lilly B, Galewsky S, Firulli AB, Schulz RA, Olson EN (1994). D-MEF2: a MADS box transcription factor expressed in differentiating mesoderm and muscle cell lineages during Drosophila embryogenesis. Proc Natl Acad Sci U S A.

[46] Shore P, Sharrocks AD (1995). The MADS-box family of transcription factors. Eur J Biochem.

[47] David R, Wedlich D (2001). PCR-based RNA probes: a quick and sensitive method to improve whole mount embryo in situ hybridizations. Biotechniques.

[48] Janssen R, Andersson E, Betnér E, Bijl S, Fowler W, Höök L, Leyhr J, Mannelqvist A, Panara V, Smith K, Tiemann S (2018). Embryonic expression patterns and phylogenetic analysis of panarthropod sox genes: insight into nervous system development, segmentation and gonadogenesis. BMC Evol Biol.

[49] Lartillot N, Lespinet O, Vervoort M, Adoutte A (2002). Expression pattern of Brachyury in the mollusc Patella vulgata suggests a conserved role in the establishment of the AP axis in Bilateria. Development.

[50] Holstien K, Rivera A, Windsor P, Ding S, Leys SP, Hill M, Hill A (2010). Expansion, diversification, and expression of T-box family genes in Porifera. Dev Genes Evol.

[51] Ahn D, You KH, Kim CH (2012). Evolution of the tbx6/16 subfamily genes in vertebrates: insights from zebrafish. Mol Biol Evol.

[52] Sebé-Pedrós A, Ariza-Cosano A, Weirauch MT, Leininger S, Yang A, Torruella G, Adamski M, Adamska M, Hughes TR, Gómez-Skarmeta JL, Ruiz-Trillo I (2013). Early evolution of the T-box transcription factor family. Proc Natl Acad Sci U S A.

[53] Sebé-Pedrós A, Ruiz-Trillo I (2017). Evolution and classification of the T-Box transcription factor family. Curr Top Dev Biol.

[54] Okkema PG (2017). The remarkably diverse family of T-Box Factors in caenorhabditis elegans. Curr Top Dev Biol.

[55] Borner J, Rehm P, Schill RO, Ebersberger I, Burmester T (2014). A transcriptome approach to ecdysozoan phylogeny. Mol Phylogenet Evol.

[56] Panfilio KA (1865). Chuva de Sousa Lopes SM The extended analogy of extraembryonic development in insects and amniotes. Philos Trans R Soc Lond B Biol Sci.

[57] Treffkorn S, Kahnke L, Hering L, Mayer G (2018). Expression of NK cluster genes in the onychophoran Euperipatoides rowelli: implications for the evolution of NK family genes in nephrozoans. EvoDevo.

[58] Mayer G, Whitington PM, Sunnucks P, Pflüger HJ (2010). A revision of brain composition in Onychophora (velvet worms) suggests that the tritocerebrum evolved in arthropods. BMC Evol Biol.

[59] Mayer G (2006). Origin and differentiation of nephridia in the Onychophora provide no support for the Articulata. Zoomorphology.

[60] Servetnick MD, Steinworth B, Babonis LS, Simmons D, Salinas-Saavedra M, Martindale MQ (2017). Cas9-mediated excision of Nematostella brachyury disrupts endoderm development, pharynx formation and oral-aboral patterning. Development.

[61] Fritzenwanker JH, Uhlinger KR, Gerhart J, Silva E, Lowe CJ (2019). Untangling posterior growth and segmentation by analyzing mechanisms of axis elongation in hemichordates. Proc Natl Acad Sci U S A.

[62] Herrmann BG, Labeit S, Poustka A, King TR, Lehrach H (1990). Cloning of the T gene required in mesoderm formation in the mouse. Nature.

[63] Smith JC, Price BM, Green JB, Weigel D, Herrmann BG (1991). Expression of a Xenopus homolog of Brachyury (T) is an immediate-early response to mesoderm induction. Cell.

[64] Wilson V, Manson L, Skarnes WC, Beddington RS (1995). The T gene is necessary for normal mesodermal morphogenetic cell movements during gastrulation. Development.

[65] Wilson V, Beddington R (1997). Expression of T protein in the primitive streak is necessary and sufficient for posterior mesoderm movement and somite differentiation. Dev Biol.

[66] Gross JM, McClay DR (2001). The role of Brachyury (T) during gastrulation movements in the sea urchin Lytechinus variegatus. Dev Biol.

[67] Yamada A, Martindale MQ, Fukui A, Tochinai S (2010). Highly conserved functions of the Brachyury gene on morphogenetic movements: insight from the early-diverging phylum Ctenophora. Dev Biol.

[68] Croce J, Lhomond G, Gache C. Expression pattern of Brachyury in the embryo of the sea urchin *Paracentrotus lividus*. Dev Genes Evol. 2001;211(12):617–9. 10.1007/s00427-001-0200-5. 10.1007/s00427-001-0200-5 11819120

[69] Rast JP, Cameron RA, Poustka AJ, Davidson EH (2002). Brachyury Target genes in the early sea urchin embryo isolated by differential macroarray screening. Dev Biol.

[70] Wilkinson DG, Bhatt S, Herrmann BG (1990). Expression pattern of the mouse T gene and its role in mesoderm formation. Nature.

[71] Amacher SL, Draper BW, Summers BR, Kimmel CB (2002). The zebrafish T-box genes no tail and spadetail are required for development of trunk and tail mesoderm and medial floor plate. Development.

[72] Martín-Durán JM, Janssen R, Wennberg S, Budd GE, Hejnol A (2012). Deuterostomic development in the protostome Priapulus caudatus. Curr Biol.

[73] Singer JB, Harbecke R, Kusch T, Reuter R, Lengyel JA (1996). Drosophila brachyenteron regulates gene activity and morphogenesis in the gut. Development.

[74] Shinmyo Y, Mito T, Uda T, Nakamura T, Miyawaki K, Ohuchi H, Noji S (2006). brachyenteron is necessary for morphogenesis of the posterior gut but not for anteroposterior axial elongation from the posterior growth zone in the intermediate-germband cricket Gryllus bimaculatus. Development.

[75] Berns N, Kusch T, Schröder R, Reuter R (2008). Expression, function and regulation of Brachyenteron in the short germband insect Tribolium castaneum. Dev Genes Evol.

[76] Hertzler PL, Wei J, Droste AP, Yuan J, Xiang J (2018). Penaeid shrimp brachyury: sequence analysis and expression during gastrulation. Dev Genes Evol.

[77] Bruce AEE, Winklbauer R (2020). Brachyury in the gastrula of basal vertebrates. Mech Dev.

[78] Yasuo H, Lemaire P. A two-step model for the fate determination of presumptive endodermal blastomeres in *Xenopus embryos*. Curr Biol. 1999;9(16):869–79. 10.1016/s0960-9822(99)80391-1 10469589

[79] Xanthos JB, Kofron M, Wylie C, Heasman J (2001). Maternal VegT is the initiator of a molecular network specifying endoderm in Xenopus laevis. Development.

[80] Clements D, Friday RV, Woodland HR (1999). Mode of action of VegT in mesoderm and endoderm formation. Development.

[81] Clements D, Woodland HR (2003). VegT induces endoderm by a self-limiting mechanism and by changing the competence of cells to respond to TGF-beta signals. Dev Biol.

[82] Li HY, Bourdelas A, Carron C, Gomez C, Boucaut JC, Shi DL (2006). FGF8, Wnt8 and Myf5 are target genes of Tbx6 during anteroposterior specification in Xenopus embryo. Dev Biol.

[83] Ban H, Yokota D, Otosaka S, Kikuchi M, Kinoshita H, Fujino Y, Yabe T, Ovara H, Izuka A, Akama K, Yamasu K, Takada S, Kawamura A (2019). Transcriptional autoregulation of zebrafish tbx6 is required for somite segmentation. Development.

[84] Belgacem MR, Escande ML, Escriva H, Bertrand S (2011). Amphioxus Tbx6/16 and Tbx20 embryonic expression patterns reveal ancestral functions in chordates. Gene Expr Patterns.

[85] Pérez O, Benítez MS, Nath K, Heasman J, Del Pino EM, Elinson RP (2007). Comparative analysis of Xenopus VegT, the meso-endodermal determinant, identifies an unusual conserved sequence. Differentiation.

[86] Wehn AK, Farkas DR, Sedlock CE, Subedi D, Chapman DL (2020). Functionally distinct roles for T and Tbx6 during mouse development. Biol Open..

[87] Chapman DL, Agulnik I, Hancock S, Silver LM, Papaioannou VE (1996). Tbx6, a mouse T-Box gene implicated in paraxial mesoderm formation at gastrulation. Dev Biol.

[88] Nikaido M, Kawakami A, Sawada A, Furutani-Seiki M, Takeda H, Araki K (2002). Tbx24, encoding a T-box protein, is mutated in the zebrafish somite-segmentation mutant fused somites. Nat Genet.

[89] Horn T, Panfilio KA (2016). Novel functions for Dorsocross in epithelial morphogenesis in the beetle Tribolium castaneum. Development.

[90] Makita R, Mizuno T, Koshida S, Kuroiwa A, Takeda H (1998). Zebrafish wnt11: pattern and regulation of the expression by the yolk cell and No tail activity. Mech Dev.

[91] Heisenberg CP, Tada M, Rauch GJ, Saúde L, Concha ML, Geisler R, Stemple DL, Smith JC, Wilson SW (2000). Silberblick/Wnt11 mediates convergent extension movements during zebrafish gastrulation. Nature.

[92] Andre P, Song H, Kim W, Kispert A, Yang Y (2015). Wnt5a and Wnt11 regulate mammalian anterior-posterior axis elongation. Development.

[93] Sinha T, Lin L, Li D, Davis J, Evans S, Wynshaw-Boris A, Wang J (2015). Mapping the dynamic expression of Wnt11 and the lineage contribution of Wnt11-expressing cells during early mouse development. Dev Biol.

[94] Van Itallie ES, Field CM, Mitchison TJ, Kirschner MW (2023). Dorsal lip maturation and initial archenteron extension depend on Wnt11 family ligands. Dev Biol.

[95] Bolognesi R, Beermann A, Farzana L, Wittkopp N, Lutz R, Balavoine G, Brown SJ, Schröder R. Tribolium Wnts: evidence for a larger repertoire in insects with overlapping expression patterns that suggest multiple redundant functions in embryogenesis. Dev Genes Evol. 2008;218(3–4):193–202. 10.1007/s00427-007-0170-3. 10.1007/s00427-007-0170-3 PMC3206738 18392880

[96] Hayden L, Schlosser G, Arthur W. Functional analysis of centipede development supports roles for Wnt genes in posterior development and segment generation. Evol Dev. 2015;17(1):49–62. 10.1111/ede.12112 25627713

[97] Janssen R, Le Gouar M, Pechmann M, Poulin F, Bolognesi R, Schwager EE, Hopfen C, Colbourne JK, Budd GE, Brown SJ, Prpic NM, Kosiol C, Vervoort M, Damen WG, Balavoine G, McGregor AP. Conservation, loss, and redeployment of Wnt ligands in protostomes: implications for understanding the evolution of segment formation. BMC Evol Biol. 2010;10:374. 10.1186/1471-2148-10-374. 10.1186/1471-2148-10-374 PMC3003278 21122121

[98] Janssen R, Pechmann M, Turetzek N. A chelicerate Wnt gene expression atlas: novel insights into the complexity of arthropod Wnt-patterning. Evodevo. 2021;12(1):12. 10.1186/s13227-021-00182-1. 10.1186/s13227-021-00182-1 PMC8579682 34753512

[99] Somorjai IML, Martí-Solans J, Diaz-Gracia M, Nishida H, Imai KS, Escrivà H, Cañestro C, Albalat R (2018). Wnt evolution and function shuffling in liberal and conservative chordate genomes. Genome Biol.

[100] Martí-Solans J, Godoy-Marín H, Diaz-Gracia M, Onuma TA, Nishida H, Albalat R, Cañestro C (2021). Massive gene loss and function shuffling in appendicularians stretch the boundaries of chordate Wnt family evolution. Front Cell Dev Biol.

[101] Hogvall M, Schönauer A, Budd GE, McGregor AP, Posnien N, Janssen R (2014). Analysis of the Wnt gene repertoire in an onychophoran provides new insights into the evolution of segmentation. EvoDevo.

[102] Probst S, Arnold SJ (2017). Eomesodermin-at dawn of cell fate decisions during early embryogenesis. Curr Top Dev Biol.

[103] Tagawa K, Humphreys T, Satoh N (2000). T-Brain expression in the apical organ of hemichordate tornaria larvae suggests its evolutionary link to the vertebrate forebrain. J Exp Zool.

[104] Maruyama YK (2000). A sea cucumber homolog of the mouse T-Brain-1 is expressed in the invaginated cells of the early gastrula in Holothuria leucospilota. Zoolog Sci.

[105] Shoguchi E, Satoh N, Maruyama YK (2000). A starfish homolog of mouse T-brain-1 is expressed in the archenteron of Asterina pectinifera embryos: possible involvement of two T-box genes in starfish gastrulation. Dev Growth Differ.

[106] Satoh G, Takeuchi JK, Yasui K, Tagawa K, Saiga H, Zhang P, Satoh N (2002). Amphi-Eomes/Tbr1: an amphioxus cognate of vertebrate Eomesodermin and T-Brain1 genes whose expression reveals evolutionarily distinct domain in amphioxus development. J Exp Zool.

[107] Horton AC, Gibson-Brown JJ (2002). Evolution of developmental functions by the Eomesodermin, T-brain-1, Tbx21 subfamily of T-box genes: insights from amphioxus. J Exp Zool.

[108] Fuchikami T, Mitsunaga-Nakatsubo K, Amemiya S, Hosomi T, Watanabe T, Kurokawa D, Kataoka M, Harada Y, Satoh N, Kusunoki S, Takata K, Shimotori T, Yamamoto T, Sakamoto N, Shimada H, Akasaka K (2002). T-brain homologue (HpTb) is involved in the archenteron induction signals of micromere descendant cells in the sea urchin embryo. Development.

[109] Minemura K, Yamaguchi M, Minokawa T (2009). Evolutionary modification of T-brain (tbr) expression patterns in sand dollar. Gene Expr Patterns.

[110] Baxendale S, Davison C, Muxworthy C, Wolff C, Ingham PW, Roy S (2004). The B-cell maturation factor Blimp-1 specifies vertebrate slow-twitch muscle fiber identity in response to Hedgehog signaling. Nat Genet.

[111] Livi CB, Davidson EH (2006). Expression and function of blimp1/krox, an alternatively transcribed regulatory gene of the sea urchin endomesoderm network. Dev Biol.

[112] Ng T, Yu F, Roy S (2006). A homologue of the vertebrate SET domain and zinc finger protein Blimp-1 regulates terminal differentiation of the tracheal system in the Drosophila embryo. Dev Genes Evol.

[113] Nakamura T, Extavour CG (2016). The transcriptional repressor Blimp-1 acts downstream of BMP signaling to generate primordial germ cells in the cricket Gryllus bimaculatus. Development.

[114] Kanai-Azuma M, Kanai Y, Gad JM, Tajima Y, Taya C, Kurohmaru M, Sanai Y, Yonekawa H, Yazaki K, Tam PP, Hayashi Y (2002). Depletion of definitive gut endoderm in Sox17-null mutant mice. Development.

[115] Zhang C, Basta T, Fawcett SR, Klymkowsky MW (2005). SOX7 is an immediate-early target of VegT and regulates Nodal-related gene expression in Xenopus. Dev Biol.

[116] Bonatto Paese CL, Leite DJ, Schönauer A, McGregor AP, Russell S (2018). Duplication and expression of Sox genes in spiders. BMC Evol Biol.

[117] Leptin M (1991). twist and snail as positive and negative regulators during Drosophila mesoderm development. Genes Dev.

[118] Jiang J, Kosman D, Ip YT, Levine M (1991). The dorsal morphogen gradient regulates the mesoderm determinant twist in early Drosophila embryos. Genes Dev.

[119] Sommer RJ, Tautz D (1994). Expression patterns of twist and snail in Tribolium (Coleoptera) suggest a homologous formation of mesoderm in long and short germ band insects. Dev Genet.

[120] Handel K, Basal A, Fan X, Roth S (2005). Tribolium castaneum twist: gastrulation and mesoderm formation in a short-germ beetle. Dev Genes Evol.

[121] Yamazaki K, Akiyama-Oda Y, Oda H (2005). Expression patterns of a twist-related gene in embryos of the spider Achaearanea tepidariorum reveal divergent aspects of mesoderm development in the fly and spider. Zoolog Sci.

[122] Domsch K, Schröder J, Janeschik M, Schaub C, Lohmann I (2021). The hox transcription factor ubx ensures somatic myogenesis by suppressing the mesodermal master regulator twist. Cell Rep.

[123] Kraus F, Haenig B, Kispert A (2001). Cloning and expression analysis of the mouse T-box gene Tbx18. Mech Dev.

[124] Beaster-Jones L, Horton AC, Gibson-Brown JJ, Holland ND, Holland LZ (2006). The amphioxus T-box gene, AmphiTbx15/18/22, illuminates the origins of chordate segmentation. Evol Dev.

[125] Begemann G, Gibert Y, Meyer A, Ingham PW (2002). Cloning of zebrafish T-box genes tbx15 and tbx18 and their expression during embryonic development. Mech Dev.

[126] Yamada A, Pang K, Martindale MQ, Tochinai S (2007). Surprisingly complex T-box gene complement in diploblastic metazoans. Evol Dev.

[127] Schulreich SM, Salamanca-Díaz DA, Zieger E, Calcino AD, Wanninger A (2022). A mosaic of conserved and novel modes of gene expression and morphogenesis in mesoderm and muscle formation of a larval bivalve. Org Divers Evol.

[128] Minguillón C, Garcia-Fernàndez J (2002). The single amphioxus Mox gene: insights into the functional evolution of Mox genes, somites, and the asymmetry of amphioxus somitogenesis. Dev Biol.

[129] Kozin VV, Filimonova DA, Kupriashova EE, Kostyuchenko RP (2016). Mesoderm patterning and morphogenesis in the polychaete Alitta virens (Spiralia, Annelida): Expression of mesodermal markers Twist, Mox, Evx and functional role for MAP kinase signaling. Mech Dev.

[130] Beer J, Technau GM, Campos-Ortega JA (1987). Lineage analysis of transplanted individual cells in embryos of Drosophila melanogaster: IV commitment and proliferative capabilities of mesodermal cells. Roux’s Arch Dev Biol.

[131] Bate M. 1993 The mesoderm and its derivatives, In: M. Bate, A. Martinez Arias (eds) The Development of Drosophila melanogaster, Cold Spring Harbor Laboratory Press, pp 1013–1090

[132] Nguyen HT, Bodmer R, Abmayr SM, McDermott JC, Spoerel NA (1994). D-mef2: a Drosophila mesoderm-specific MADS box-containing gene with a biphasic expression profile during embryogenesis. Proc Natl Acad Sci U S A.

[133] Taylor MV, Beatty KE, Hunter HK, Baylies MK (1995). Drosophila MEF2 is regulated by twist and is expressed in both the primordia and differentiated cells of the embryonic somatic, visceral and heart musculature. Mech Dev.

[134] Price AL, Patel NH (2008). Investigating divergent mechanisms of mesoderm development in arthropods: the expression of Ph-twist and Ph-mef2 in Parhyale hawaiensis. J Exp Zool B Mol Dev Evol.

[135] Janssen R, Damen WG (2008). Diverged and conserved aspects of heart formation in a spider. Evol Dev.

[136] Leite DJ, Schönauer A, Blakeley G, Harper A, Garcia-Castro H, Baudouin-Gonzalez L, Wang R, Sarkis N, Günther AN, Poojitha Koka VS, Nathan J. Kenny NJ, Turetzek N, Pechmann M, Solana J, McGregor AP. An atlas of spider development at single-cell resolution provides new insights into arthropod embryogenesis bioRxiv 2022.06.09.495456; doi: 10.1101/2022.06.09.495456 10.1186/s13227-024-00224-4 PMC11083766 38730509

[137] Schulz RA, Chromey C, Lu MF, Zhao B, Olson EN (1996). Expression of the D-MEF2 transcription in the Drosophila brain suggests a role in neuronal cell differentiation. Oncogene.

[138] Bush JO, Maltby KM, Cho ES, Jiang R (2003). The T-box gene Tbx10 exhibits a uniquely restricted expression pattern during mouse embryogenesis. Gene Expr Patterns.

[139] Dastjerdi A, Robson L, Walker R, Hadley J, Zhang Z, Rodriguez-Niedenführ M, Ataliotis P, Baldini A, Scambler P, Francis-West P (2007). Tbx1 regulation of myogenic differentiation in the limb and cranial mesoderm. Dev Dyn.

[140] Greulich F, Rudat C, Kispert A (2011). Mechanisms of T-box gene function in the developing heart. Cardiovasc Res.

[141] Martín-Durán JM, Romero R (2011). Evolutionary implications of morphogenesis and molecular patterning of the blind gut in the planarian Schmidtea polychroa. Dev Biol.

[142] Janssen R (2014). Gene expression suggests double-segmental and single-segmental patterning mechanisms during posterior segment addition in the beetle Tribolium castaneum. Int J Dev Biol.

[143] Schaub C, Nagaso H, Jin H, Frasch M (2012). Org-1, the Drosophila ortholog of Tbx1, is a direct activator of known identity genes during muscle specification. Development.

[144] Nomaru H, Liu Y, De Bono C, Righelli D, Cirino A, Wang W, Song H, Racedo SE, Dantas AG, Zhang L, Cai CL, Angelini C, Christiaen L, Kelly RG, Baldini A, Zheng D, Morrow BE (2021). Single cell multi-omic analysis identifies a Tbx1-dependent multilineage primed population in murine cardiopharyngeal mesoderm. Nat Commun.

[145] Venuti JM, Gan L, Kozlowski MT, Klein WH (1993). Developmental potential of muscle cell progenitors and the myogenic factor SUM-1 in the sea urchin embryo. Mech Dev.

[146] Chen L, Krause M, Sepanski M, Fire A (1994). The Caenorhabditis elegans MYOD homologue HLH-1 is essential for proper muscle function and complete morphogenesis. Development.

[147] Zhang JM, Chen L, Krause M, Fire A, Paterson BM (1999). Evolutionary conservation of MyoD function and differential utilization of E proteins. Dev Biol.

[148] Wardle FC (2019). Master control: transcriptional regulation of mammalian Myod. J Muscle Res Cell Motil.

[149] Passamaneck YJ, Hejnol A, Martindale MQ (2015). Mesodermal gene expression during the embryonic and larval development of the articulate brachiopod Terebratalia transversa. EvoDevo.

[150] Venuti JM, Goldberg L, Chakraborty T, Olson EN, Klein WH (1991). A myogenic factor from sea urchin embryos capable of programming muscle differentiation in mammalian cells. Proc Natl Acad Sci U S A.

[151] Janssen R (2011). Diplosegmentation in the pill millipede Glomeris marginata is the result of dorsal fusion. Evol Dev.

