# Supplementary figures and images for: New insights into mesoderm and endoderm development, and the nature of the onychophoran blastopore
Source: Front Zool. 2024 Jan 25;21:2. doi: 10.1186/s12983-024-00521-7 (PMC10809584; doi:10.1186/s12983-024-00521-7)

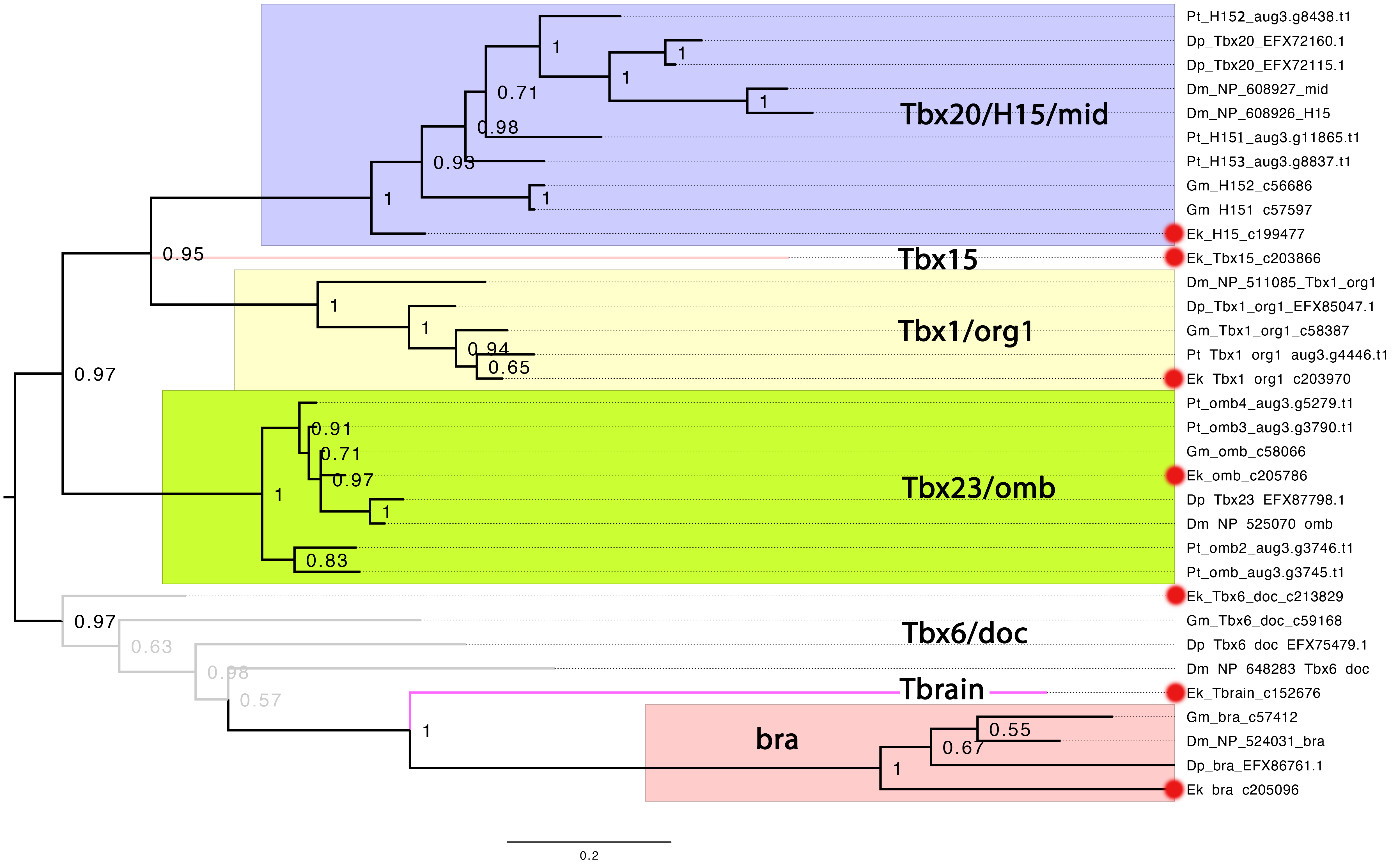

Supplement: Supplementary file 1 — Additional file1: Phylogenetic tree of arthropod T-box genes. Bayesian analyses. The scale bar represents the amino acid substitutions rate per site. Species and accession numbers are listed in Supplementary File 7. See text for further information. [file 12983_2024_521_MOESM1_ESM.tif]

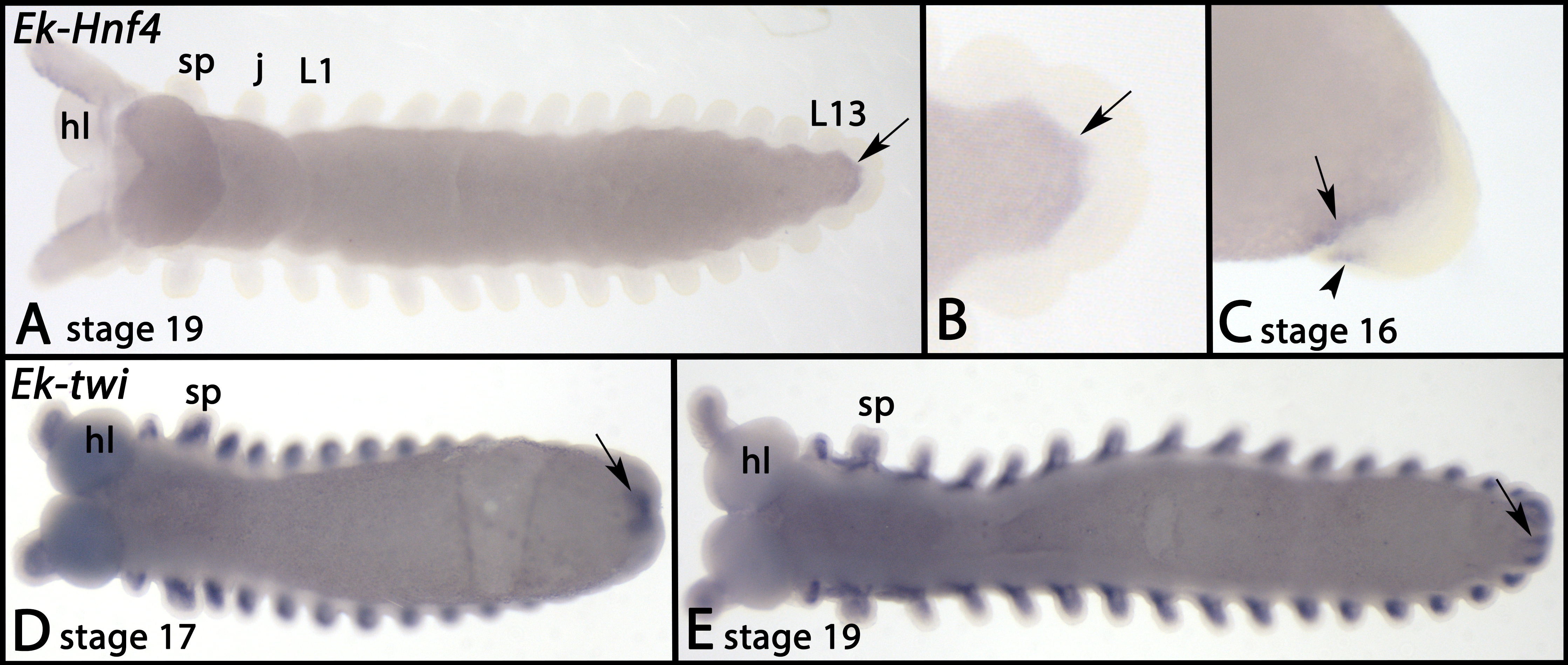

Supplement: Supplementary file 3 — Additional file 3: Late Expression of Hnf4 and twi. In all panels, anterior is to the left, ventral views (except panel C that shows a lateral view). Arrows point to posterior expression associated with de novo formation of endoderm and mesoderm. Abbreviations as in Figure 2. [file 12983_2024_521_MOESM3_ESM.tif]
